# Identification and Characterization of Nep1-Like Proteins From the Grapevine Downy Mildew Pathogen *Plasmopara viticola*

**DOI:** 10.3389/fpls.2020.00065

**Published:** 2020-02-13

**Authors:** Stefan Schumacher, Katrin Grosser, Ralf Thomas Voegele, Hanns-Heinz Kassemeyer, René Fuchs

**Affiliations:** ^1^ Section of Phytopathology and Diagnosis, Department of Biology, State Institute for Viticulture and Enology, Freiburg, Germany; ^2^ Department of Phytopathology, Institute of Phytomedicine (360), Faculty of Agricultural Sciences, University of Hohenheim, Stuttgart, Germany

**Keywords:** *Plasmopara viticola*, grapevine, Nep1, Necrosis and Ethylene inducing peptide 1 (Nep1)-like proteins, necrosis, ethylene, *Vitis*, innate immunity

## Abstract

The obligate biotrophic oomycete *Plasmopara viticola* causes tremendous problems in viticulture by evoking grapevine downy mildew. *P. viticola*, like other plant pathogens, achieves infection by suppression of plant innate immunity by secretion of effector molecules into its host plant. An ever-expanding family of proteins with effector-like characteristics is formed by the “Necrosis and Ethylene inducing peptide 1 (Nep1)-like proteins” (NLPs). NLPs can be divided into two groups by their ability to induce necrosis. While cytotoxic NLPs may act as virulence factors for a necrotrophic or hemibiotrophic plant pathogen, the role of non-cytotoxic NLPs is so far unknown. In this study, we identified eight independent NLPs in *P. viticola* and selected three for functional analysis. While one was identified as a putative pseudo gene, two contain all so far described critical key elements for necrosis formation except for an N-terminal signal peptide. Further characterization revealed that none of the putative necrosis elicitors was able to actually induce necrosis, neither in several susceptible or resistant *Vitis* species nor in the dicot model plant *Nicotiana benthamiana*. This inability exists independently of the presence or absence of a signal peptide. However, any possible mechanism for the suppression of the ability to induce necrosis *in planta* was not detected. Interestingly, expression analysis of the presumed pseudo gene revealed remarkable differences between pure sporangia solution and sporangia in the presence of leaf material. To our knowledge, this is the first report of this kind of regulation that suggests an important function of so far nonfunctional “pseudo” NLP genes during the first hours of infection.

## Introduction

Grapevine downy mildew belongs to the most challenging problems in viticulture (*Vitis* spp.) and demands expensive and laborious efforts from winegrowers all over the globe (Gessler, 2011).The causal agent of this disease is the obligate biotrophic oomycete *Plasmopara viticola* (Berk. & Curt. *ex.* de Bary), which was accidentally introduced into Europe from North America in the late 19^th^ century. Indigenous American grapevine species representing the natural host plant of this pathogen were under continuous infection pressure which led to a highly effective form of plant innate immunity (Boso and Kassemeyer, 2008). The prevalent common grape vine (*Vitis vinifera*) in Europe was never challenged to develop such a system for its protection. Today *P. viticola* is controlled by frequent fungicide application over the whole vegetation period. Increasing fungicide resistance as well as growing concerns about the environmental impact resulting in governmental regulations calls for the development of alternative and sustainable strategies for *P. viticola* control (Corio-Costet, 2012).

Over the course of evolution, plants have developed a complex system of defenses which enables them to withstand the majority of potentially dangerous microorganisms in their environment. Besides some rather unspecific mechanical and chemical preformed barriers they benefit from a very specific system, the plant innate immunity (Jones and Dangl, 2006). The first reactions in this immunity happen during the so called PAMP-triggered immunity (PTI). During PTI a broad range of defense responses is induced after recognition of pathogen-associated molecular patterns (PAMPs) throughout pattern recognition receptors (PRR) on the cell surface (Jones and Dangl, 2006). Defense reactions include for example the production of reactive oxygen species (ROS), closing of the stomata, callose deposition in the cell wall or the production of several phytoalexins or pathogenesis related (PR) proteins (Meng and Zhang, 2013). Adapted pathogens have developed several ways to circumvent PTI by using specialized proteins which are usually referred to as effectors (Jones and Dangl, 2006). During the so called effector triggered susceptibility (ETS), these pathogen secreted effectors contribute to the pathogens' virulence which is why they are also called virulence factors (Jones and Dangl, 2006). On the other hand plants themselves have developed ways to recognize effectors or the targets influenced by these effectors. This consequently leads to effector triggered immunity (ETI), which includes similar defense reactions like PTI, but often resulting in a rapid cell death at the infection site, referred to as hypersensitive response (HR) (Coll et al., 2011). Former virulence factors of the pathogen are now unfavorable for the pathogen and are therefore called avirulence factors (Jones and Dangl, 2006). During their coevolution plants and pathogens have constantly developed new forms of ETS and ETI which is why this is often referred to as an evolutionary arms race (Boller and He, 2009).

An ever-expanding group of pathogen-derived proteins with effector like characteristics are the “necrosis- and ethylene-inducing peptide 1 (NEP1)-like proteins” (NLPs) (Pemberton and Salmond, 2004). Proteins of this family are highly conserved over all kingdoms of microorganisms and share highest similarities to cytotoxic actinoporins as well as to fungal lectins (Ottmann et al., 2009). The majority of these proteins share structural characteristics which are directly linked to their necrosis-inducing ability. One such characteristic structural element are conserved cysteine residues. Cysteine residues are responsible for disulfide bond formation which has a critical impact on the tertiary structure and therefore the function of a protein (Betz, 1993). After the substitution of one cysteine residue necrosis-inducing *Phytophthora* protein 1 (NPP1) purified from *Phytophthora parasitica* was no longer able to induce necrosis when applied directly onto parsley (*Petroselinum crispum*) leaves (Fellbrich et al., 2002). Another key element for cytotoxicity is the hepta peptide motif GHRHDWE. This motif is essentially involved in shaping a cavity on the protein surface which was shown to be critical for the ability to induce necrosis in several studies (Ottmann et al., 2009; Chen et al., 2018). Since it was shown that membrane disruption induced by NLPs takes place from the apoplastic side, it is assumed that at least necrosis formation mediated by these proteins requires the presence of a signal peptide (Qutob et al., 2006). This necessity was clearly demonstrated with two NLPs from *Phytophthora sojae* (NLP*_Ps_*) and *P. parasitica* (NLP*_Pp_*) (Qutob et al., 2006). Both showed no cytotoxic activity when transiently expressed without the signal peptide (SP^-^) in sugar beet (*Beta vulgaris*). However, transient expression of type 1 NLP*_Ps_* (SP^-^) in *N. benthamiana* led to, although delayed compared with the wild-type protein, clear necrosis formation, even when produced inside the endoplasmic reticulum (ER) (Oome and Van den Ackerveken, 2014). Interestingly, transient expression of the type 2 NLP*_Pcc_* from *Pectobacterium carotovora* did not induce any necrosis when targeted to the ER or expressed without signal peptide, suggesting different modes of action for these two NLPs and type 1 and type 2 NLPs in general (Oome and Van den Ackerveken, 2014). Besides these cytotoxic elements, NLPs also carry a 24 amino acid peptide (nlp24) which is recognized as a PAMP in several plants (Böhm et al., 2014; Oome et al., 2014). nlp24 is able to induce several reactions of PTI and is recognized by the receptor complex RLP23-SOBIR1-BAK1 in several species within the *Brassicaceae* and *Asteraceae* (Albert et al., 2015).

The first NLP (NEP1) was discovered in the causal agent of the vascular wilt, *Fusarium oxysporum* (Bailey, 1995). Since then, NLPs were found in all kinds of prokaryotic and eukaryotic microorganisms; however, the majority is known from plant pathogenic oomycetes and fungi (Gijzen and Nürnberger, 2006; Oome and Van den Ackerveken, 2014). The highest copy numbers were so far identified in the genus *Phytophthora* with for example 33 known NLPs and 37 pseudogenes in *P. sojae* (Dong et al., 2012). Necrotrophic pathogens, like *Pythium ultimum* (six NLPs) show considerably fewer copy numbers (Lévesque et al., 2010). For biotrophic plant pathogens NLPs were reported for example from *Plasmopara halstedii* (19 NLPs, 1 pseudogene) and *Hyaloperonospora arabidopsidis* (14 NLPs and 15 pseudogenes) (Cabral et al., 2012; Sharma et al., 2015). However, functional analysis of NLPs from an obligate biotroph was performed only for several NLPs from *H. arabidopsidis* (Cabral et al., 2012).

NLPs can be divided into two general groups. Group one consists of proteins with a strong ability to induce necrosis. Hemibiotrophic pathogens express these proteins during the switch from biotrophic to necrotrophic lifestyle (Alkan et al., 2015). Several studies showed that these NLPs contribute strongly to the virulence of their pathogens (Amsellem et al., 2002; Santhanam et al., 2013). Group two, on the other hand, consists of non-cytotoxic NLPs. Most of these proteins are expressed during the earliest infection stages (Cabral et al., 2012; Dong et al., 2012). These NLPs are reported from several hemibiotrophic and biotrophic plant pathogens (Cabral et al., 2012; Dong et al., 2012; Zhou et al., 2012). The function of these NLPs is so far unknown. It can be assumed that obligate biotrophic pathogens lost or modified their cytotoxic NLPs during evolution, however, the high number of remaining NLPs without the ability to induce necrosis indicates an important additional role, especially during early infection stages. The objective of this work was to identify and characterize NLPs from *P. viticola*. Specifically, their ability to induce necrosis was analyzed, and their function in the pathogen-plant interaction was elucidated.

Identification of NLP coding genes was done by analysis of the three published sequenced genomes of *P. viticola*. For in depth molecular and biochemical characterization, we selected three NLPs of *P. viticola*. The corresponding genes were sequenced in isolates collected from middle Europe and Israel to analyze their conservation. Gene expression studies were carried out under various experimental setups to reflect the whole course of infection. NLPs were expressed transiently in *N. benthamiana* to study necrosis-inducing ability, subcellular localization as well as potential homo- or heterodimerization. Furthermore we analyzed the ability to induce necrosis in several *Vitis* spp. by vacuum infiltration of recombinant proteins.

## Materials and Methods

### Identification and *In Silico* Analysis of *P. viticola NLPs* (*PvNLPs*)

#### Gene Identification and Analysis

*NLP* genes of *P. viticola* (*PvNLPs*) were identified by mega blast queries (National Center for Biotechnology Information (NCBI), Bethesda MD, USA) against the three known genome assemblies of the isolates INRA-PV221 (INRA, France), JL-7-2 (China Agriculture University, China), and PvitFEM01 (Fondazione Edmund Mach, Italy) using the peptide “AIMYSWYFPKDMPSTDFGHRHDWE” (**Supplemental Table 1**). DNA- and protein assemblies were performed with Clustal Omega (Sievers et al., 2011). Signal peptide prediction was performed with SignalP 5 (Armenteros et al., 2019). Non-classical secretion was predicted with SecretomeP 2.0a (Bendtsen et al., 2005). LOGOs were created with Weblogo after manual removal of signal peptides from the proteins. Colors were set at KRH: green, DE: blue, ST: red, VLAGI: orange, NQ: purple and C: RGB 8C8C8C. Phylogenetic analysis was performed with the sequences of the eight PvNLPs and the 230 oomycete NLPs used by Oome and Van den Ackerveken (2014) according to their protocol. Alignments for maximum likelihood trees were created using mafft (LINSi) after manual removal of the signal peptide (Katoh et al., 2019). Positions present in less than 75% of the sequences were removed manually from the alignment. Phylogenetic analysis was performed with PhyML (v3.0, WAG model, four substitution rate categories, estimated proportion of invariable sites, estimated gamma distribution parameters, and a robustness assessment of 100 bootstraps) using Phylogeny.fr (Dereeper et al., 2008). The phylogenetic tree was designed with FigTree v1.4.4 (Rambaut, 2018). Disulfide bond prediction was performed with Disulfind (Ceroni et al., 2006).

#### Sequencing of *P. viticola* Isolates

Genomic DNA from 36 field isolates and seven single sporangia lines obtained in equal proportions from susceptible and tolerant grape varieties from vine yards in Europe (Germany, France) and the Middle East (Israel) have been analyzed. The precise list of the analyzed cultivars as well as their year and location of sampling is given in **Supplemental Table 2**. Genomic DNA was provided from collaborators or isolated from infected leaves with the Plant DNA Kit (Analytik Jena AG, Jena, Germany). Genomic DNA from sporangia or mycelium of *P. viticola* and *Phytophthora infestans* was isolated with the Bacteria & Fungi DNA Kit (Analytik Jena AG) according to the manufacturer's instructions. Amplification was carried out with the PCRBIO HiFi Polymerase (PCR Biosystems Ltd, London, UK) with the corresponding primers (**Supplemental Table 3**). PCR products were cleaned up from agarose gels with the NucleoSpin^®^ Gel and PCR Clean-up Kit (Macherey-Nagel, Düren, Germany) and cloned “blunt end” into the plasmid pUC19 (Life Technologies, Carlsbad CA, USA). Plasmids from three positive colonies were isolated by alkaline lysis as published before (Sambrook et al., 1989) and sequenced with the primers pUC-F and pUC-R. Sequence analysis was performed with Vector NTI Advance 11.5.3 (Life Technologies).

### Gene Expression Analysis

#### Plant Inoculation and Pathogen Propagation

All inoculation experiments as well as the propagation of *P. viticola* were carried out with the highly susceptible common grape vine (*Vitis vinifera* subsp. *vinifera*) cultivar Mueller-Thurgau (clone FR3, rootstock 125 AA) (Boso and Kassemeyer, 2008). Inoculation experiments were carried out with the 3^rd^ to 5^th^ leaf counting from the tip due to their highest susceptibility (Steimetz et al., 2012). Infiltration experiments were carried out with *V. vinifera* cultivar Mueller-Thurgau as well as with American and Asian wild grapes *Vitis riparia*, *Vitis rupestris,* and *Vitis amurensis* and the model plant *Nicotiana benthamiana*.

*P. viticola* propagation was carried out every 5 to 8 days by spraying of a sporangia solution (40,000 sporangia/ml) which was obtained by rinsing of infected leaves with autoclaved tap water.

#### Expression Experiments

Relative expression of *PvNLPs* was analyzed with infected leaf disks at seven different time points representing the whole course of infection beginning with the release of the zoospores from sporangia until the formation of new sporangia. Time points were defined according to Unger et al. (2007) as follows: t_0_ (right after rinsing of sporangia) was set as reference point; t_1_ (1 h after application) represents the release of zoospores from sporangia; t_2_ (3 h after application) represents the stage of encysted zoospores which already begin to form first germ tubes. Six hours after application (t_3_) germ tubes already enter the substomatal cavity, first haustoria are visible under the microscope; 24 h (t_4_) after application, branching of the hyphae inside the host plant is clearly visible; 72 h (t_5_) after application hyphae spread over a whole intercostal field; 96 h (t_6_) after application the oomycete begins to infect adjacent intercostal fields; 144 h (t_7_) after application almost the whole leaf disk is filled with oomycete structures, on the leaf disks surface mycelium and sporangia are visible with the naked eye.

Leaf disks were produced with a cork borer (Ø 15 mm) and inoculated with 50-µl droplets of a sporangia solution (50.000/ml). Two leaf disks including the droplets were pooled for one sample and frozen in liquid nitrogen. In addition, two leaf disks per time point were fixated in 80% EtOH and stained with Aniline Blue for fluorescence microscopy as described (Kiefer et al., 2002).

Expression experiments in sporangia solution were carried out on samples representing four different time points: t_1_ 15 min after rinsing, t_2_ 30 min, t_3_ 60 min; t_4_ 150 _min_. t_0_ was used as a reference immediately after rinsing. Sporangia solutions were obtained by rinsing of 5 to 8 days old, freshly sporulating material from infected leaves. The solution was adjusted to 50.000 sporangia/ml. 1.5 ml of the solution was centrifuged (13,000*g*, 10 min, RT), the supernatant was discarded and the pellet immediately transferred to liquid nitrogen.

RNA from infected leaf disks was isolated with the Spectrum™ Plant Total RNA Kit (Sigma-Aldrich, Steinheim, Germany) according to the manufacturer's instructions. Isolation from sporangia pellets was performed with the GeneMATRIX Universal RNA Purification Kit (Roboklon GmbH, Berlin, Germany). RNA concentrations were leveled, and cDNA was synthesized using an oligo(dT)_18_ – primer and the MMLV High Performance Reverse Transcriptase (Epicentre, Madison WI, USA). qPCR was performed with the SensiMix™ SYBR^®^ & Fluorescein Kit (Bioline, Luckenwalde, Germany) and the corresponding primers (**Supplemental Table 3**). Relative, normalized expression values were calculated with the double delta Ct method (Pfaffl, 2001). The housekeeping gene *Actin* was used as a reference. All expression experiments were carried out in three different biological replicates (one replicate corresponds to one independent sporangia solution). Every biological replicate consisted of three technical replicates.

### Transient Expression of *PvNLPs*

#### Transformation of *N. benthamiana*

Transformation of *N. benthamiana* was accomplished with the help of *Agrobacterium tumefaciens* strain GV3101 with Ti-plasmid pMP90RK (Koncz and Schell, 1986). Subcellular localization as well as cytotoxicity assays were performed with the plasmids pAMPAT/pAMMCS (Lipka et al., 2005; Fuchs et al., 2016) with or without the coding sequence for a fluorescent dye, respectively.

For cytotoxicity assays NLP genes were amplified from genomic DNA of *P. viticola* with the corresponding primers (**Supplemental Table 3**) as described. Since the original *PvNLP1* is truncated because of a single guanine deletion, a *full-length PvNLP1* (*PvNLP1_FL_*) was produced and analyzed in addition. *PvNLP1_FL_* was produced by adding the missing base to the ORF of *PvNLP1* with the corresponding primers (**Supplemental Table 3**) referring to a site-specific mutagenesis protocol published before (Zheng et al., 2004). The necrosis inducing NLP *PiNPP1.1* from *P. infestans* was used as a positive control. PCR products and plasmids were digested with the restriction enzymes *Xho*I and *Eco*RI and ligated with the T4 DNA Ligase (Thermo Fisher Scientific, Dreieich, Germany).

For subcellular localization, NLPs were tagged to green fluorescent protein (GFP) and red fluorescent protein (RFP), respectively. NLPs were analyzed in experiments with an N-terminal as well as with a C-terminal fluorescent protein. Tagging of GFP/RFP to the N-terminus was obtained by *Xho*I/*Not*I digestion and ligation of plasmid and PCR product. For C-terminal tags the enzymes *Not*I/*Eco*RI were used.

Transformation was performed using the protocol by Ma et al. (2012). All transformations were conducted in co-infiltration with the silencing suppressor p19 (Lakatos et al., 2004). Agrobacteria without any NLP construct but with p19 were infiltrated as a negative control in the cytotoxicity assays. Infiltrated leaves were observed for necrosis over 14 days. Cell death was visualized by Trypan Blue staining as published (Ma et al., 2012).

Subcellular localization was observed under a fluorescent microscope (Axio Imager.Z1, Carl Zeiss AG, Oberkochen, Germany) three days after *A. tumefaciens* infiltration. Excitation of the corresponding leaf areas took place at 475 nm for GFP (Filter Set 38 HE, Carl Zeiss AG) and at 532 nm for RFP (Filter Set 43 HE, Carl Zeiss AG).

#### Domain Swapping

The influence of two different N-terminal domains on the necrosis-inducing ability of *Pv*NLPs was studied by performing domain-swaps as before published for NLPs from *H. arabidopsidis* (Cabral et al., 2012). In the first chimeric construct the signal peptide (SP) from *Pi*NPP1.1 (consisting of the first 19 amino acids) was ligated directly to the N-terminus of the *Pv*NLPs. In the second chimeric construct the whole N-terminal domain ending with the second conserved cysteine residue (first 70 amino acids of *Pv*NLP1-3) was chosen and exchanged with the corresponding domain from *Pi*NPP1.1 (first 83 amino acids).

For the ligation of the mentioned domains to the N-terminus of *Pv*NLPs the corresponding domains were amplified as described above to produce PCR products with either *Pae*I or *Kpn*I restriction sites. After clean-up, the ligated product was used as a template for a second PCR to add restriction sites for cloning into plasmids pAMPAT and pAMMCS, respectively.

#### Co-Immunoprecipitation

Homo- or heterodimerization of *Pv*NLPs was studied in co-immunoprecipitation experiments. For this purpose GFP-/RFP- tagged NLPs were co-expressed in *N. benthamiana*. Protein extraction was carried out as described (Moffett et al., 2002) with minor modifications. Precipitation was performed with GFP-antibody coated agarose beads (GFP-Trap^®^; ChromoTek, Martinsried, Germany) as recommended by the manufacturer. Precipitated proteins were analyzed by immunoblot analysis under strong denaturing conditions in Tris-Glycine buffer, containing 2.1% (w/v) SDS and 5% (v/v) β-mercaptoethanol. Samples were separated using Criterion TGX Stain-Free Precast Gels (Bio-Rad, Munich, Germany). Blotting was performed with the Trans-Blot Turbo Transfer System (Bio-Rad) and Trans-Blot Turbo 0.2 µm PVDF membranes (Bio-Rad) according to manufacturer's manual. Membranes were probed with anti-GFP or anti-RFP antibody (ChromoTek).

### Cytotoxicity Assays in Different *Vitis* spp.

#### Heterologous Expression and Vacuum Infiltration

To produce recombinant protein the corresponding genes were amplified as described above, cloned into the vector pET24a(+) (Novagen-Merck Bioscience Ltd, Nottingham, UK) and expressed in *E.coli* BL21-CodonPlus^®^(DE3)-RIL (Stratagene – Agilent Technologies, Santa Clara, CA, USA) as recommended by the manufacturer. Proteins were extracted under denaturing conditions as stated in the Protino Ni-IDA manual with minor modifications. Final clean-up of His-tagged NLPs was done with the Protino Ni-IDA 1000 Kit (Macherey-Nagel).

Refolding of the proteins into their natural conformation was achieved by step-wise substitution of the 8 M urea buffer with Hepes or PBS buffer, respectively. For the dialysis Slide-A-Lyzer G2 dialysis cassettes (7K MWCO, 3ml, Thermo Fisher Scientific) were used. Before dialysis protein solutions were adjusted to an OD_595_ of 0.7. Two milliliters of the protein solution were injected into a cassette and put into 250-ml dialysis puffer at a temperature of 25°C under constant stirring. Buffer was replaced every 24 h to step-wise reduce the urea concentration. To avoid precipitation, different buffers with increasing glycerol concentrations and decreasing urea concentrations were used. *Pv*NLP1 and *Pv*NLP3 dialysis worked best with Hepes buffer (20 mM, pH 7) while for *Pv*NLP2 and *Pi*NPP1.1 PBS (pH 8) was more convenient. Urea and glycerol concentrations in the different dialysis buffers were: 6 M urea and 10% (v/v) glycerol, 4 M urea and 17.5% (v/v) glycerol, 2 M urea and 25% (v/v) glycerol, 1 M urea and 37.5% (v/v) glycerol, and finally 50% (v/v) glycerol without urea. Due to the high glycerol concentrations, proteins could be stored at −20°C until further usage. Proteins were validated under denaturing conditions by immunoblot analysis as described above. For probing an anti-His antibody (Invitrogen) was diluted 1:5.000 in Tris-buffered saline with Tween 20 (TBST) (20 mM Tris-HCl pH 7.5, 500 mM NaCl, 0.1% Tween 20) with 0.2% skimmed milk powder.

Infiltration of *Vitis* leaf disks was performed with a desiccator. Leaf disks were obtained as described above and put upside down into a 12-well cell culture plate. The non-ionic wetting agent Break-Thru S240 (Evonik, Essen, Germany) was added to the protein solutions (0.01% (v/v)) to improve the infiltration results. Negative pressure was generated and maintained for 2 min and afterward released slowly. Infiltrated leaf disks were transferred to a water agar plate (1% (w/v)) and observed for 14 days under a 16-h light/8-h dark rhythm at 25°C.

## Results

### Identification of the *Plasmopara viticola NLP* Gene Family

For the identification of *NLPs* in *P. viticola* blast searches were performed with the peptide AIMYSWYFPKDMPSTDFGHRHDWE from the “*Plasmopara viticola* NPP1-like protein” (GenBank: KC243261) against the three available genome assemblies INRA-PV221, JL-7-2, and PvitFEM01. Eight different *NLP* coding genes were identified and named *PvNLP1*–*PvNLP8* (*Plasmopara viticola* necrosis and ethylene inducing peptide 1- like protein 1–8) (**Supplemental Table 1**).

The size of *Pv*NLP2 – *Pv*NLP8 ranges from 223 to 271 amino acids (**Table 1**). *Pv*NLP1 carries a deletion of a single nucleotide which leads to a frameshift and an early Stop-codon. Therefore, *Pv*NLP1 is assumed to be a pseudogene with a length of 345 bp and a size of 114 amino acids. *Pv*NLP1, *Pv*NLP2, and *Pv*NLP3 as well as *Pv*NLP4, *Pv*NLP5, and *Pv*NLP8 share a high degree of homology and presumably developed through gene duplication from two ancestral genes. A strong N-terminal signal peptide was predicted for *Pv*NLP5, *Pv*NLP6, *Pv*NLP7, and *Pv*NLP8. For the other four proteins SecP scores were calculated, indicating a high possibility for non-classical secretion (*Pv*NLP1: 0.86; *Pv*NLP2: 0.91; *Pv*NLP3: 0.90; *Pv*NLP4 0.81). The peptide nlp24, is present in all *Pv*NLPs, except *Pv*NLP1 - although with several alterations in amino acid sequence.

**Table 1:** Specifications of PvNLPs.

**ID**	**Length (aa)**	**Mw (kDa)**	**pI**	**Type**	**Signal-peptide**	**QQQ/TPAP**	**Disulfide bridges**	**Ca^2+^-site**	**Cation-binding pocket**
PvNLP1	114	12.25	7.75		no (0.0027)	no	2 (43–69) and (92–101)	no	no
PvNLP2	223	25.05	6.63	1	no (0.0024)	no	1 (43–69)	no	yes
PvNLP3	223	25.19	8.49	1	no (0.003)	no	1 (43–69)	no	yes
PvNLP4	271	31.11	7.21	1a	no (0.4696)	no	1 (91–116)	no	no
PvNLP5	239	27.64	7.04	1a	yes (0.9994)	no	1 (56–81)	no	no
PvNLP6	267	29.99	8.76	1a	yes (0.8795)	no	1 (71–96)	no	no
PvNLP7	241	27.17	9.37	1a	yes (0.9949)	no	1 (58–84)	no	no
PvNLP8	242	28.01	7.11	1a	yes (0.9982)	no	1 (62–87)	no	no

**Figure 1:**
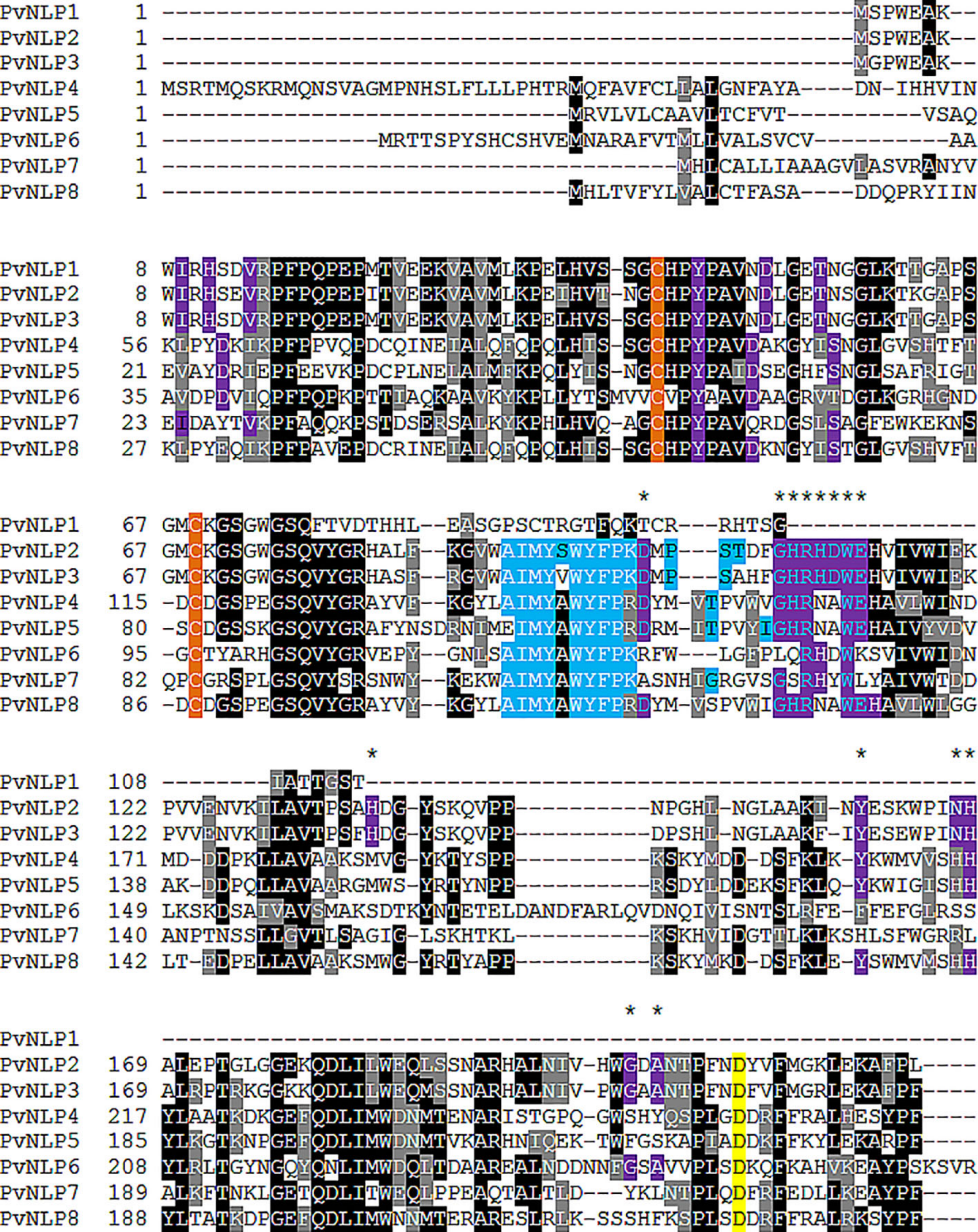
Sequence characteristics of PvNLPs: Amino acid sequence alignment of all identified *Pv*NLPs. Purple color indicates amino acids conserved in type 1 NLPs including the heptapeptide motif, asterisk indicate the amino acids suggested to be critical for cytotoxicity (D93, G100, H101, R102, H103, D104, W105, E106, H128, Y151, N158, H159, G193, and A195 according to Oome and Van den Ackerveken (2014). Orange color highlights the conserved cysteine residues; blue color indicates amino acids identical to the MAMP nlp24 published by Oome et al. (2014); yellow color indicates amino acids conserved in type 1a NLPs; grey color indicates amino acids with similar chemical properties.

**Figure 2:**
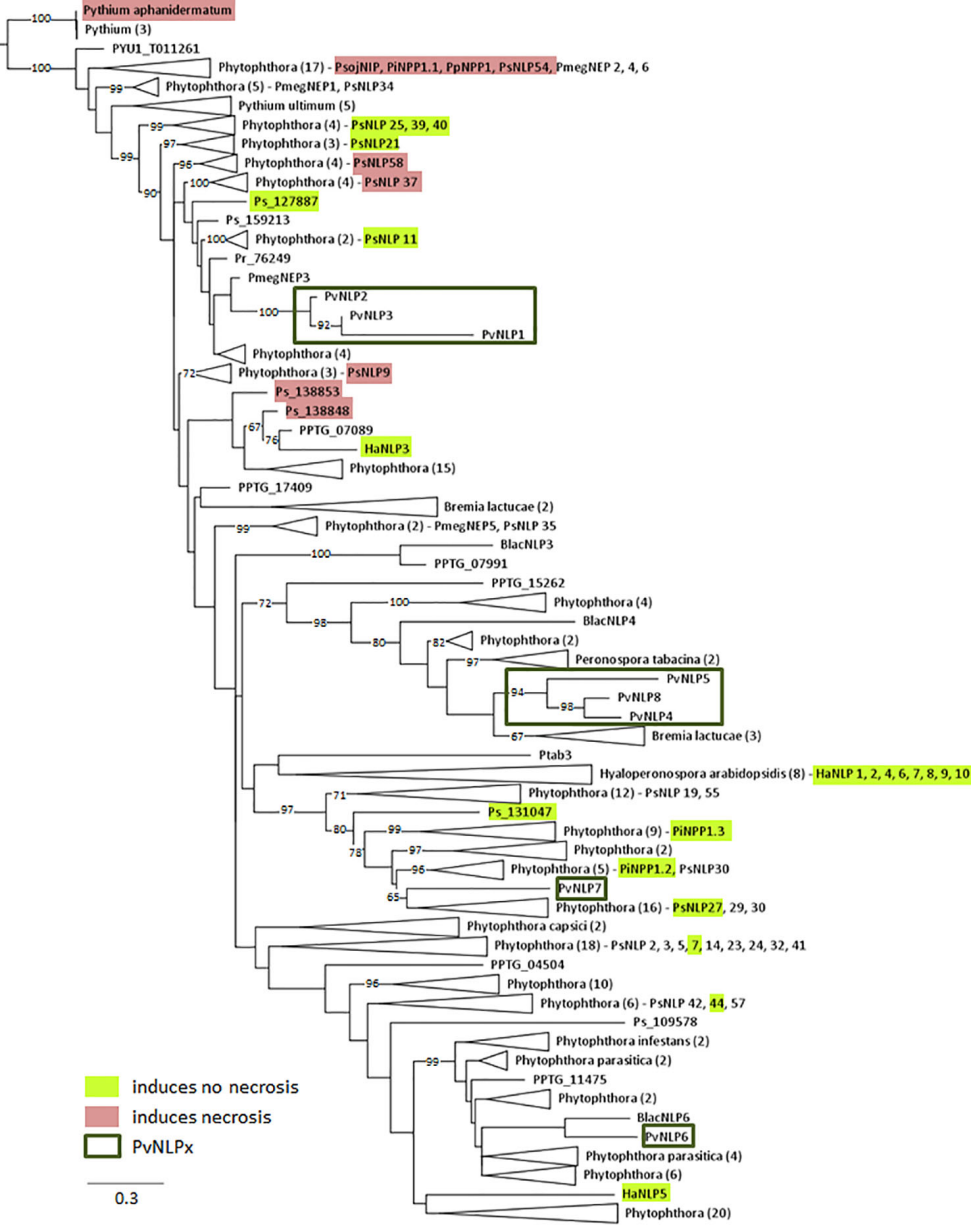
Phylogenetic relationship of 238 oomycete NLPs including NLPs of *P. viticola* (*Pv*NLP): The maximum likelihood tree was constructed according to the one constructed by Oome and Van den Ackerveken (2014). NLP shown: *Pmeg*NEP1-6 (Bae et al., 2005); *Pi*NPP1.1, *Pi*NPP1.2, *Pi*NPP1.3 (Kanneganti et al., 2007); *Pp*NPP1, NLP*_Pya_*_(_ *_Pythium__aphanidermatum_*_)_ (Ottmann et al., 2009); PsojNIP, HaNLP1-10 (Cabral et al., 2012); PsNLP1, 2, 3, 5, 7, 8, 9, 11, 14, 19, 21, 23, 24, 25, 27, 29, 30, 32, 35, 37, 38, 39, 40, 41, 42, 44, 51, 54, 55, 57, 58, 59, and 60 (Dong et al., 2012). Numbers on branches indicate bootstraps (only values >60 are shown).

All identified *Pv*NLPs carry two conserved cysteine residues which classifies them as type 1 NLPs (Gijzen and Nürnberger, 2006). A complete hepta peptide (GHRHDWE) motif is only present in *Pv*NLP2 and *Pv*NLP3, whereas it is completely missing in *Pv*NLP1 due to its truncation (**Figure 1**). In order to reconstruct the relationship between *Pv*NLPs and NLPs identified in other oomycetes a phylogenetic analysis was carried out (**Figure 2**). For this purpose the eight *Pv*NLPs were compared to the 230 oomycete NLPs also used for the phylogenetic analysis by Oome and Van den Ackerveken (2014) (**Supplemental Figure 1**: LOGO of the sequences of *Pv*NLPs compared to the other 230 oomycetes).

As expected the results show that *Pv*NLPs which presumably developed from the same ancestral gene cluster together. *Pv*NLP1, *Pv*NLP2, and *Pv*NLP3 form a new branch inside a cluster of NLPs from different *Phytophthora* species. The NLPs of this cluster carry additional glutamine (Q)-rich or proline (TPAP-repeat) - rich hydrophilic regions, which are suggested targets for post-translational modification e.g. O-glycosylation (Oome and Van den Ackerveken, 2014). However *Pv*NLP1, *Pv*NLP2, and *Pv*NLP3 do not carry such domains. *Pv*NLP4, *Pv*NLP5, and *Pv*NLP8 form a branch next to three NLPs from *Bremia lactucae* (*Blac*NLPs), a downy mildew species infecting lettuce. Interestingly, only *Pv*NLP6 and *Pv*NLP7 cluster with type 1a NLPs. While *Pv*NLP6 clusters next to *Blac*NLP6, *Pv*NLP7 clusters into a branch of 45 NLPs from different *Phytophthora* NLPs including the non-cytotoxic NLPs *Pi*NPP1.2 and *Pi*NPP1.3 (Kanneganti et al., 2007). Since *Pv*NLP 4, *Pv*NLP5, *Pv*NLP8 as well as *Pv*NLP6 and *Pv*NLP7 show several mutations in the amino acids conserved in type 1 NLPs, which were described as crucial for necrosis (**Figure 1**), we categorize them as type1a NLPs. *Pv*NLP2 and *Pv*NLP3 carry an intact heptapeptide motif and all other amino acids critical for formation of a cation-binding site and are therefore type 1 NLPs.

### *Pv*NLP1, *Pv*NLP2, and *Pv*NLP3 Are Highly Conserved

Functional characterization concentrated on *Pv*NLP2 and *Pv*NLP3, which are the only *Pv*NLPs containing all crucial amino acids for the induction of innate immunity or the formation of necrosis. Disregarding its truncation, *Pv*NLP1 shares a high homology with *Pv*NLP3. Since the truncation is caused by a single nucleotide deletion, *Pv*NLP1 was included in the following experiments. Even though these three proteins are missing a strong signal peptide, they were assumed the most likely candidates out of the eight *Pv*NLPs to induce necrosis in plants.

In order to analyze the conservation of these three genes in general, we sequenced *PvNLP* genes from 43 isolates of *P. viticola*. The isolates were sampled from grapevine varieties that are either highly susceptible or resistant to grapevine downy mildew, growing on vineyards in central and southern Europe as well as in the Middle East over a period of 3 years (**Supplemental Table 2**).

The analyzed *PvNLP*s were overall highly conserved. The deletion leading to the truncation of *PvNLP1* was present in every isolate analyzed. Besides several silent mutations in all of the *PvNLP* genes (data not shown), we identified a substitution of a single Valine with a Leucine in *PvNLP1* in an isolate from the susceptible cultivar Gewuerztraminer sampled in 2012 (**Supplemental Figure 2**). In the same year we sampled an isolate from the resistant cultivar Prior, which also showed a single amino acid substitution from Threonine to Lysine in *PvNLP2* (**Supplemental Figure 3**). In the gene coding for *PvNLP3* we identified an exchange of a Valine with an Isoleucine. This substitution was present in isolates from four resistant and one susceptible cultivar collected from different vineyards located around Freiburg (Germany) in three consecutive years (**Supplemental Figure 4**). The mutation lies in a region of very high conservation between all the NLPs. However, this region was never linked to any function.

### *PvNLP1* Expression Is Strongly Induced During the First 24 h of Infection

**Figure 3:**
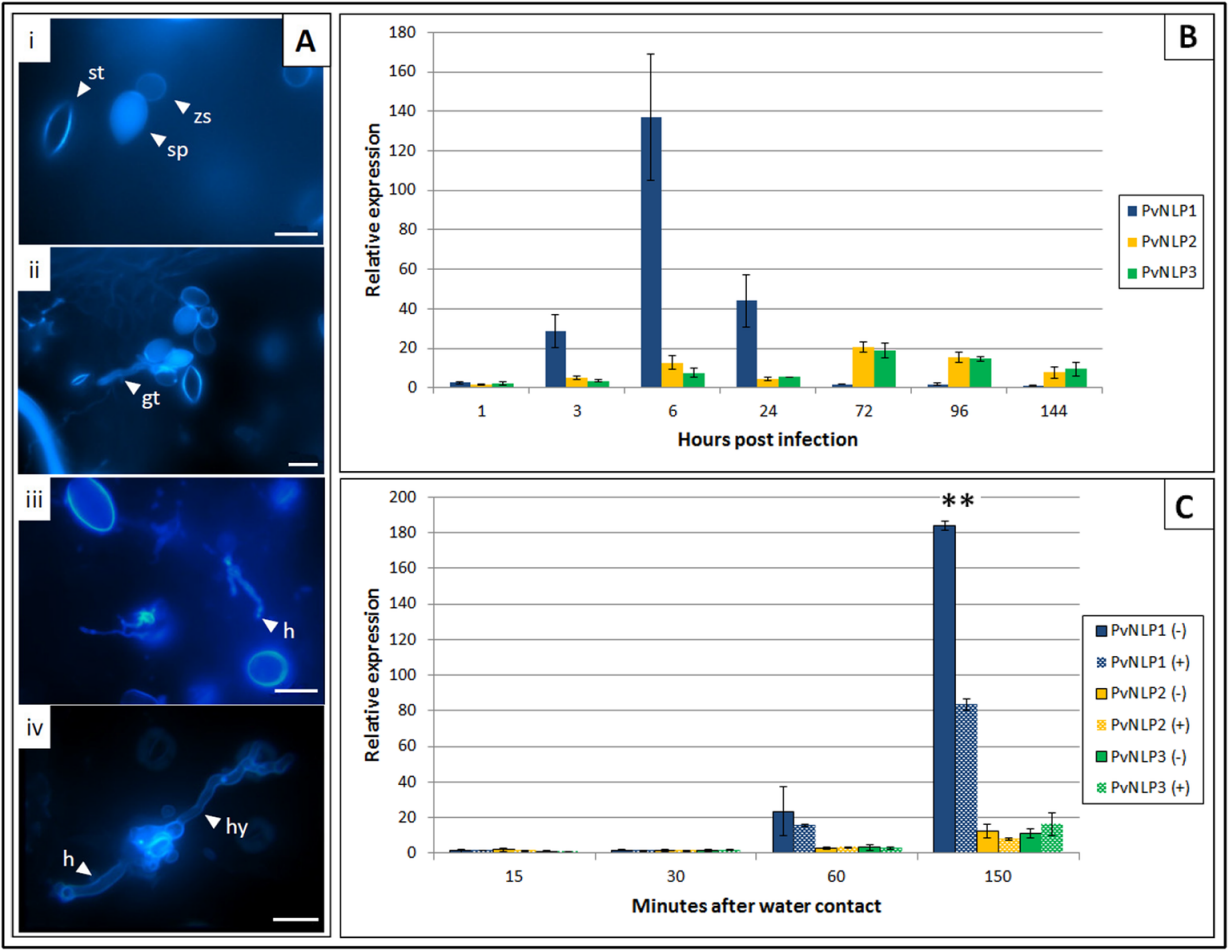
Expression of *P. viticola NLPs* 1 to 3 during infection of *V. vinifera* cv. Mueller-Thurgau: **(A)** Fluorescence microscopy pictures of Aniline Blue stained leaf disks representing different *P. viticola* stages during the infection of *V. vinifera* cv. Meuller-Thurgau: i: 1 hpi, ii: 3 hpi, iii: 6 hpi, iv: 24 hpi; gt, germ tube; h, haustoria; hy, hypha; sp, sporangium; st, stoma; zs, zoospore; scale bars = 20µM. **(B)** Relative expression of *Pv*NLP1, *Pv*NLP2, and *Pv*NLP3 on infected *V. vinifera* cv. Mueller-Thurgau leaf disks at the time points: zoospore release (1 h), penetration (3 h), first haustoria visible (6 h), branching of hyphae (24 h), 72h, 96h, and 144 h after inoculation. The displayed graph represents one of three experiments with similar results. Bars are the mean of three technical replicates ± SD. **(C)** Relative expression of *PvNLP*1, *PvNLP2,* and *PvNLP3* in a sporangia solution with (+) or without (−) presence of leaf material at the time points 15, 30, 60 (beginning of zoospore release from the sporangia), and 150 min after water contact. The displayed graph represents one of three experiments with similar results. Bars are the mean of three technical replicates ± SD. Asterisks indicate significance (p ≤ 0.01, one-way Anova).

Expression of *PvNLPs* was studied over the whole course of infection (**Figure 3B**). All genes monitored were activated shortly after contact of sporangia with water. *PvNLP2* and *PvNLP3* expression increases during the first 6 h of the infection and stays elevated until *de novo* formation of sporangia (144 hpi). By contrast, truncated *PvNLP1* shows a strong activation during the first 24 h. Levels increase from the time point on when germ tubes enter the leaf (3 hpi, **Figure 3Aii**) and the maximum expression level is reached during the formation of first haustoria (6 hpi, **Figure 3Aiii**). Thereafter, expression decreases during branching of hyphae (24 hpi, **Figure 3Aiv**) to a not detectable level during the last days of the observed period.

Expression of the analyzed *PvNLPs* in a pure sporangia solution starts one hour after first water contact of the sporangia (**Figure 3C**). Remarkably, expression of *PvNLP1* was significantly reduced when leaf strips were added to the sporangia solution and removed just before centrifugation (**Figure 3C**; **Supplemental Figure 5**).

### *Pv*NLPs Are Not Able to Induce Necrosis

To check *Pv*NLPs for their ability to induce necrosis, we transiently expressed the corresponding genes in *N. benthamiana*. In addition to *Pv*NLP1, *Pv*NLP2, and *Pv*NLP3 we infiltrated a full-length version of the truncated gene *PvNLP1* (*PvNLP1_FL(full-length)_*) to clarify if the deletion led to the loss of a possible necrosis inducing ability.

**Figure 4:**
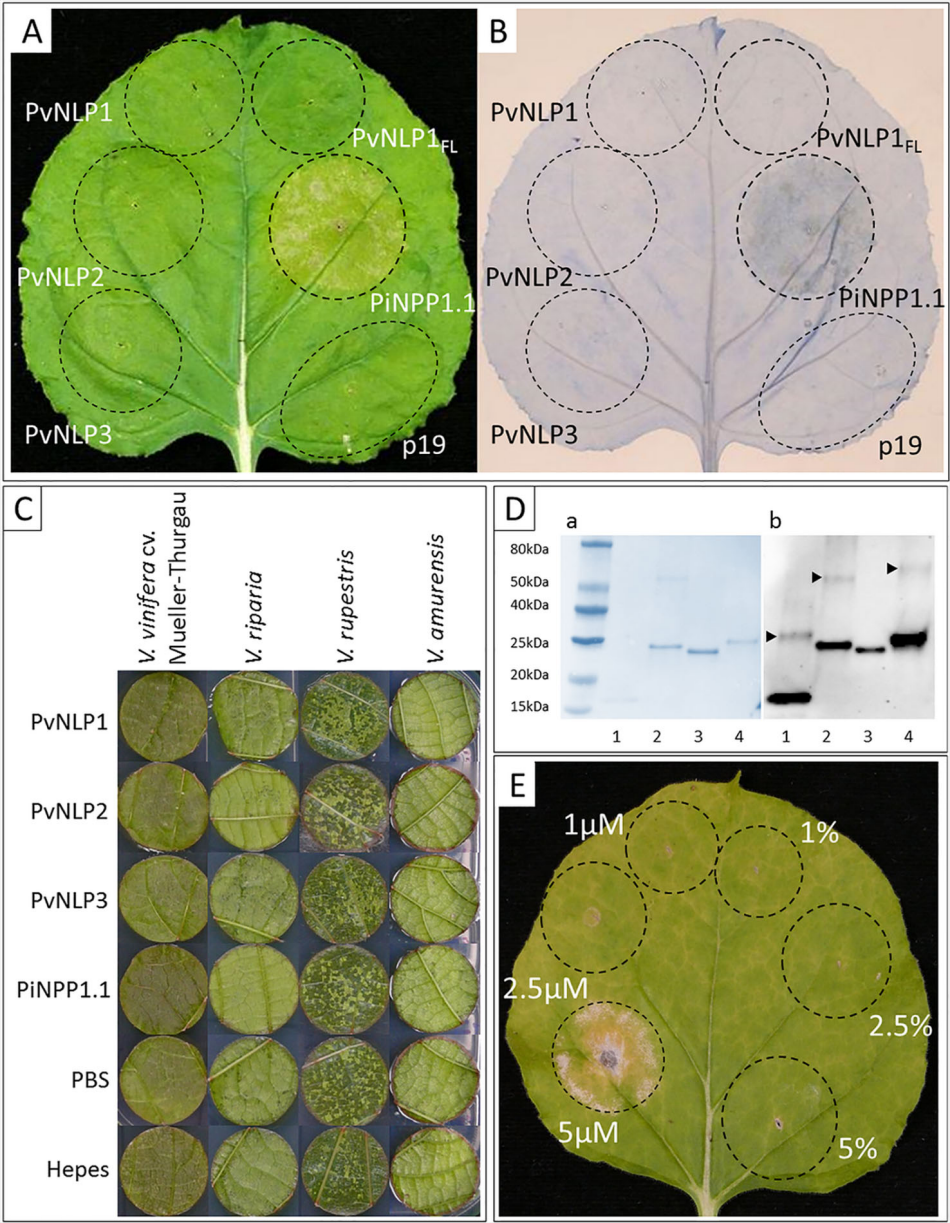
*Pv*NLP1, *Pv*NLP2, and *Pv*NLP3 are not able to induce necrosis *in planta*: **(A)** Transient expression of NLPs in a leaf of *N. benthamiana*; **(B)** Trypan Blue staining for cell death; both pictures were taken 60 h after infiltration (p19 = silencing suppressor, *PvNLP1_FL_* = full-length version of *PvNLP1*). **(C)** Vacuum-infiltration of recombinant NLPs into leaf disks of different *Vitis* species. Concentration used: 10 µM, controls represent the different protein buffers (including 10% (v/v) glycerol). The picture was taken 14 days after infiltration. **(D)** Validation of recombinant NLPs by Western-blot: 1: *Pv*NLP1, 2: *Pv*NLP2, 3: *Pv*NLP3, 4: *Pi*NPP1.1; a: Coomassie-stained blotting membrane; b: Western-Blot with anti-His antibody; arrows indicate bands representing potential homodimerization; homodimerization of *Pv*NLP3 not visible due to low concentration of dimers. **(E)** *Pi*NPP1.1 induces necrosis in *N. benthamiana* at concentrations above 5 µM (picture taken 7 days after infiltration; percentage indicate buffer controls (PBS, pH8) with respective amounts of glycerol). All pictures of infiltrated leaves or leaf disks demonstrate the results of three independent experiments with the same result.

None of the three original *Pv*NLPs (*Pv*NLP1, *Pv*NLP2 or *Pv*NLP3) nor the artificial *Pv*NLP1_FL_ was able to induce necrosis in *N. benthamiana*. Spots infiltrated with the positive control *Pi*NPP1.1 (Kanneganti et al., 2007) showed beginning chlorosis after 48 h which developed into necrotic tissue within the next 24 h (**Figure 4A**). HR was detected by Trypan Blue staining after 48 h for spots infiltrated with *Pi*NPP1.1. However, none of the spots transformed with *Pv*NLP1, *Pv*NLP2 or *Pv*NLP3 showed any hypersensitive response at any time point (**Figure 4B**).

A potential necrosis inducing activity of *Pv*NLPs was also tested in the natural host plant *V. vinifera*. For this purpose, recombinant versions of the proteins were produced by heterologous expression (predicted molecular weights: PvNLP1: 12.25 kDa; PvNLP2: 25.05 kDa; PvNLP3: 25.19 kDa; PiNPP1.1: 25.52 kDa). Verification of recombinant proteins was achieved through immunoblot analysis revealing a potential homodimerization of the used proteins in solution (**Figure 4D**). Functionality of PiNPP1.1 was validated by infiltration into *N. benthamiana* (**Figure 4E**). *Pi*NPP1.1 was infiltrated in three different concentrations (1 µM, 2.5 µM, and 5 µM). Formation of necrosis was clearly visible with a concentration of 5 µM 2 to 3 days after infiltration. Lower concentrations were not able to induce necrosis (**Figure 4E**). Furthermore, several concentrations of glycerol in the infiltration buffer were tested to exclude false positive results. Glycerol concentrations up to 10% (v/v) were tolerated by the plants, while higher concentrations led to necrosis (result not shown).

All proteins and buffers were vacuum infiltrated into leaf discs of the susceptible *V. vinifera* cultivar Mueller-Thurgau as well as into leaf disks of the resistant varieties *V. riparia*, *V. rupestris,* and *V. amurensis*. However, recombinant *Pv*NLPs were not able to induce necrosis in leaf disks of any tested *Vitis* species during the observed period of 14 days. *Pi*NPP1.1, inducing strong necrosis in *N. benthamiana*, showed no cytotoxic effect in any *Vitis* species, even if the used concentration was doubled (10 µM) (**Figure 4C**).

### Domain Swapping Between *Pi*NPP1.1 and *Pv*NLPs Does Not Restore Necrosis-Inducing Activity

**Figure 5:**
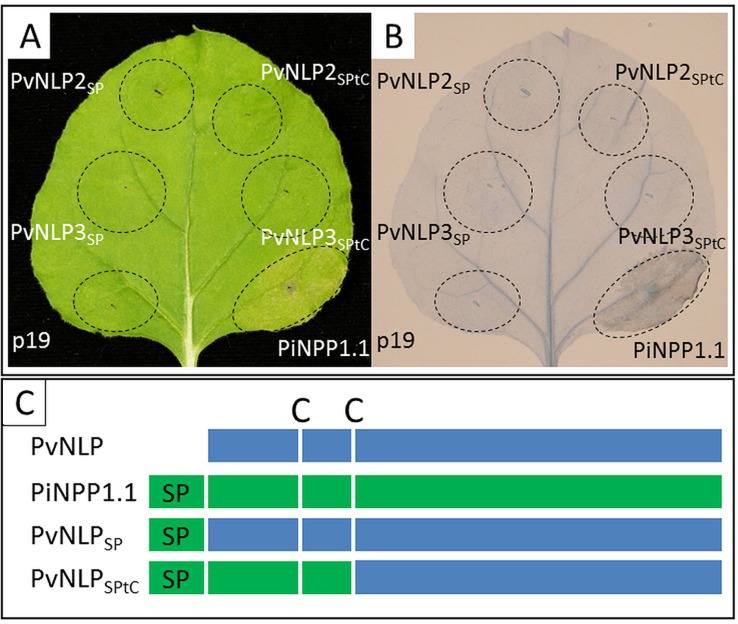
Domain-swapping does not lead to necrosis-inducing *Pv*NLPs: **(A)** Transient expression of different chimeric constructs in *N. benthamiana*; **(B)** Trypan Blue staining of cell death; both pictures were taken 60 h after infiltration (p19 = silencing suppressor). **(C)** Scheme of the created chimeric constructs (according to Cabral et al., 2012; C, cysteine; SP, signal peptide).

To analyze the influence of N-terminal domains on the ability to induce necrosis, two chimeric constructs of N-terminal domains from the strong necrosis inducing *Pi*NPP1.1 combined with non-cytotoxic *Pv*NLPs were created and transiently expressed in *N. benthamiana* (**Figure 5A, C**). All transformed leaves were observed for 14 days and a possible HR was stained with Trypan Blue (**Figure 5B**). However, none of the used chimeric constructs were able to induce necrosis in *N. benthamiana*. Neither the ligation of the N-terminal signal peptide from *Pi*NPP1.1 directly to the N-terminus of the *Pv*NLPs (*Pv*NLP_SP_) nor the substitution of the whole domain, starting at the N-terminus and ending at the second conserved cysteine (*Pv*NLP_SPtC_), had an effect on the cytotoxicity of the proteins (**Figure 5A**). An alignment of the C-termini of *Pv*NLP2 and *Pv*NLP3 with ten cytotoxic NLPs and *Ha*NLP3 revealed six amino acids which are completely different between *Pv*NLPs and the other 11 NLPs. However, an influence of these amino acids remains speculative (**Supplemental Figure 6**).

### *Pv*NLP1, *Pv*NLP2, and *Pv*NLP 3 Are Located in the Cytoplasm

**Figure 6:**
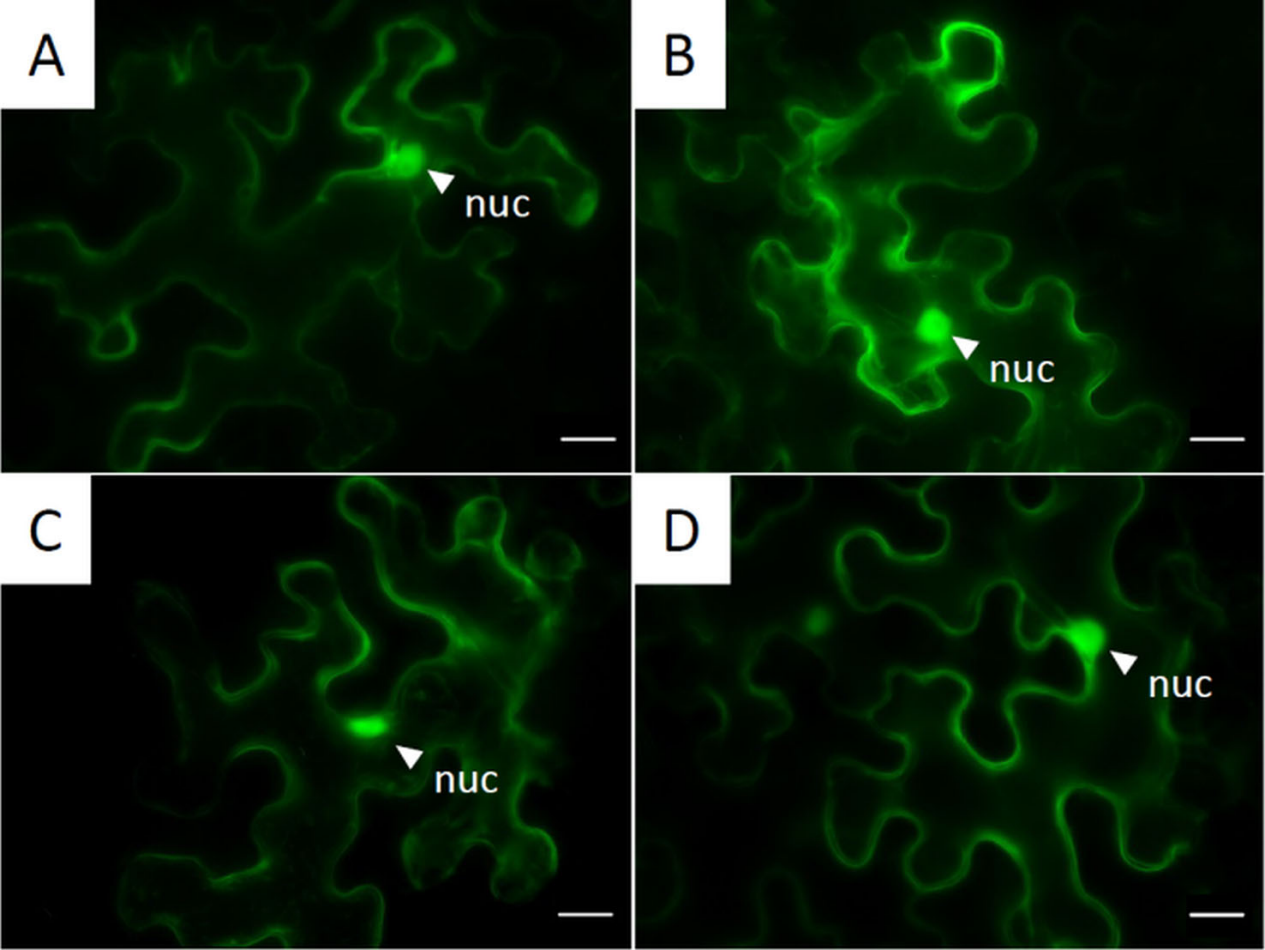
Subcellular localization of GFP-tagged NLPs inside the cytoplasm: Pictures are obtained from fluorescent microscopy of transiently expressed C-terminal GFP-labeled NLPs in *N. benthamiana* 72 h post infiltration. **(A)** *Pv*NLP1; **(B)** *Pv*NLP2; **(C)** *Pv*NLP3; **(D)**: enhanced GFP (untagged); nuc, nucleus; scale bar = 20 µm.

In order to localize *Pv*NLPs on a subcellular level, proteins were fused to fluorescent proteins and transiently expressed in *N. benthamiana*. Every analyzed *Pv*NLP-GFP showed the same pattern of localization inside the cytoplasm (**Figure 6**). Differences between C-terminal and N-terminal linked proteins were not detected (data not shown). Furthermore, we observed no differences in localization between GFP and RFP tagged NLP fusion proteins (data not shown).

### *PvNLPs* Do Not Form Oligomers *In Planta*

**Figure 7:**
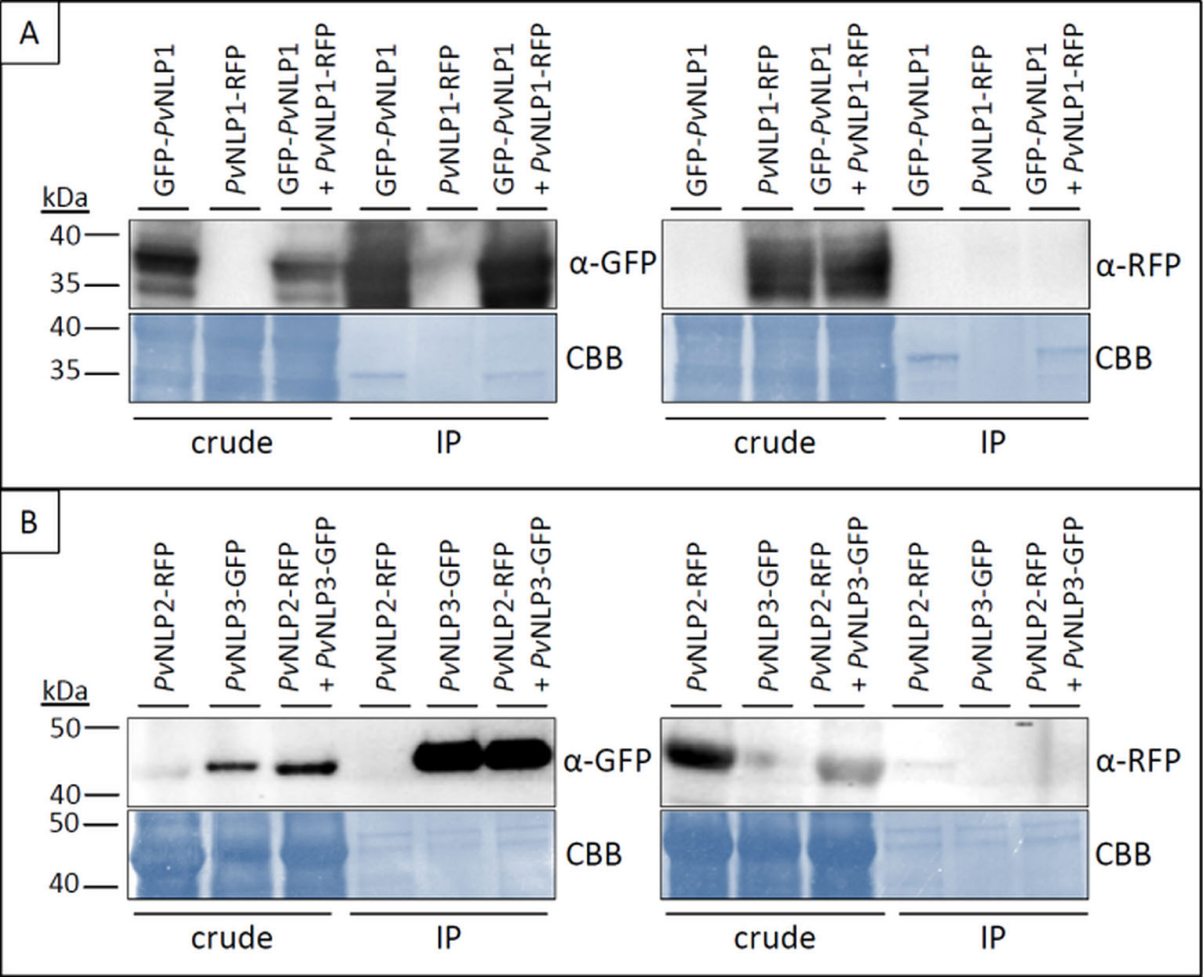
*Pv*NLPs do not form homo- or heterodimers *in planta*. Pictures show two examples of co-immunoprecipitation experiments of *Pv*NLPs labeled with a fluorescent protein. **(A)** N-terminal tagged *Pv*NLP1 in combination with C-terminal tagged *Pv*NLP1; **(B)** C-terminal tagged *Pv*NLP2 in combination with C-terminal tagged PvNLP3. Predicted size of *Pv*NLP2+RFP and *Pv*NLP3+GFP = ~ 47 kDa; predicted size of *Pv*NLP1 + GFP/RFP = ~ 36 kDa. IP, immunoprecipitation; CBB, Coomassie Brilliant Blue.

Several NLPs show the tendency to form dimers or oligomers (de Oliveira et al., 2012). Since immunoblot analysis showed that *Pv*NLPs as well as *Pi*NPP1.1 may form stable dimers in solution (**Figure 4D**) co-immunoprecipitation experiments were carried out to analyze this phenomenon *in planta* using GFP-antibody coated agarose beads (**Figure 7**).

The experiments showed no interactions between any of the tested NLPs. Neither homodimerization of a *Pv*NLP nor heterodimerization between the different *Pv*NLPs was observed. Furthermore, we were not able to detect an interaction between a NLP from *P. viticola* and *Pi*NPP1.1 (results not shown). Whether the fluorescent protein was linked to the C- or N- terminus of the NLPs or not had no influence on the results (results not shown).

## Discussion

### The *P. viticola* NLP Family

During the past 25 years NLPs were identified and characterized in several microorganisms of all kingdoms (Gijzen and Nürnberger, 2006; Oome and Van den Ackerveken, 2014). NLPs with a strong ability to induce necrosis are found in many necrotrophic or hemibiotrophic plant pathogens (Gijzen and Nürnberger, 2006). These cytotoxic NLPs are obviously beneficial during the necrotic phase of a pathogens life cycle (Amsellem et al., 2002; Santhanam et al., 2013). Contrary to the cytotoxic NLPs, there is a second, almost equally big group of non-cytotoxic NLPs found in hemibiotrophic and biotrophic microorganisms (Oome and Van den Ackerveken, 2014). The role of these NLPs is unknown.

Sequence analysis performed during this work revealed eight different NLPs in the obligate biotrophic oomycete *P. viticola*. Yin et al. (2015) performed transcriptome analyzes in two *P. viticola* isolates from China and one from Australia and indicated seven, four, and six NLPs, respectively. The three assemblies blasted during this work contained five to seven NLPs. However, none of the assemblies contained all eight *Pv*NLPs (**Supplemental Table 1**). To this point it is not sure if this divergence is isolate dependent or just caused by gaps or failures in the assembly of the different genome sequences. During this study the genes of *PvNLP1*, *PvNLP2,* and *PvNLP3* from 36 field isolates and seven single sporangia lines were sequenced (**Supplemental Table 2**). *PvNLP1*, *PvNLP2,* and *PvNLP3* were amplified in every isolate, even though *PvNLP1* was not detected during sequence analysis of the assembly from isolate JL-7-2 (China Agriculture University, China) and *PvNLP3* not in the assemblies of the isolates INRA-PV221 (INRA, France) and PvitFEM01 (Fondazione Edmund Mach, Italy). *PvNLP3* is only present in JL-7-2 and has the exact same length as *Pv*NLP2. However, both are located on the same scaffold and are clearly distinguishable with a homology of 87%, suggesting both are independent genes and not alleles.

Sequencing results obtained in this work, showed a high conservation of the different genes in *P. viticola*. However, only three colonies of every strain or isolate were picked and tested. These colonies may represent only one allele of the heterozygous organism. Furthermore, it should be taken into account that field isolates presumably represent a mixture of different strains. Nevertheless, the very low variation between the isolates collected from regions distributed over Baden-Wuerttemberg (Germany) and far distant areas in Israel indicate that *PvNLP1*, *PvNLP2* and *PvNLP3* are highly conserved.

Out of the eight NLPs from *P. viticola*, only *Pv*NLP2 and *Pv*NLP3 contain the amino acids D93, G100, H101, R102, H103, D104, E106, H128, Y151, N158, H159, G193, and A195 which were suggested to be crucial for necrosis formation (Ottmann et al., 2009; Oome and Van den Ackerveken, 2014; Chen et al., 2018). In addition we included *Pv*NLP1 since restoring of the missing guanine in its ORF leads to a full-length NLP (*Pv*NLP1_FL_) with an intact GHRHDWE motif and the amino acids mentioned above. Shortened NLPs like *Pv*NLP1 appear in high numbers in many other plant pathogens (Gijzen and Nürnberger, 2006; Cabral et al., 2012; Dong et al., 2012). Due to their truncation and the lack of several key elements described as necessary for the necrosis inducing activity, these genes are often labeled as pseudogenes. By its original definition pseudogenes are non-functional DNA domains, which arose from previously functional genes because of early stop codons or shifts in their open reading frame which are neither transcribed or translated (Li et al., 1981). However, several studies demonstrate that some pseudogenes are expressed and fulfill new tasks beyond their original function (Balakirev and Ayala, 2004). Since the function of non-cytotoxic NLPs is so far unknown, shortened versions should not per se classify as non-functional just because they lost key elements for the ability to induce necrosis. The loss of their cytotoxic character may actually be beneficial depending on the time point of expression or the lifestyle of the pathogen.

### *PvNLP1* Is Expressed During Early Infection Stages

Besides their ability to induce necrosis, these two groups of NLPs are, with few exceptions, also distinguishable with regard to their expression. Necrosis inducing NLPs are expressed at time points when hemibiotrophic pathogens switch their lifestyle from biotrophic to necrotrophic and necrotic pathogens start to kill plant material. These proteins contribute significantly to the virulence of their hosts. Non-cytotoxic NLPs, like the NLPs from *H. arabidopsidis* or the NLPs from *P. sojae* are often already expressed during very early stages of the infection and in some cases keep elevated during the whole course of infection at rather low levels (Cabral et al., 2012; Dong et al., 2012). *PvNLP1* is upregulated during the first 24 h of infection. However, afterwards *PvNLP1* expression was no longer detectable, while *PvNLP2* and *PvNLP3* are expressed over the whole infection period. Similar levels of expression were reported for the non-cytotoxic NLPs of *H. arabidopsidis* (Cabral et al., 2012). *HaNLP1*, *HaNLP3*, *HaNLP8,* and *HaNLP10* showed highest expression levels within the first 12 h after the infection whereas the genes *HaNLP2*, *HaNLP4,* and *HaNLP9* are expressed during the whole observed period. Necrosis inducing NLPs from hemibiotrophic plant pathogens like *PiNPP1.1* or *VdNLP1* and *VdNLP2* from *Verticillium dahliae* are expressed at significantly later time points. *PiNPP1.1* expression starts to increase three to four days after inoculation (Kanneganti et al., 2007). Expression levels of *VdNLP1* and *VdNLP2* increase 4 days after infection and reach their maximum 12 days after infection (Santhanam et al., 2013).

Expression ratios of *PvNLP1, PvNLP2,* and *PvNLP3* measured in germinating sporangia increased as soon as zoospores were released. Interestingly, *PvNLP1* expression was significantly decreased if leaf material was added to the sporangia solution. Even though it is still unknown if *PvNLP1* is coding for a functional protein or a pseudogene, this result suggests a mechanisms for the regulation of *PvNLP1* expression before the first physical contact with the leaf. This regulation suggests a mechanism for the recognition of present plant material by *P. viticola*. The principle underlying this recognition may be chemotaxis. Chemotaxis is for example described in detail for the symbiotic colonization of plant roots by bacteria (Bais et al., 2006). An example for chemotaxis between grapevine and the pathogen *P. viticola* may be the detection of stomata by the zoospores throughout volatile compounds like nonanal and decanal released from the substomatal cavity (Schroeder, 2010). However, additional experiments must clarify if this recognition is specific for the interaction between *V. vinifera* and *P. viticola*. Further analysis of the underlying volatile compounds may be promising—assuming differences between resistant and susceptible *Vitis* species—regarding future disease management and resistance breeding.

### *Pv*NLP1, *Pv*NLP2, and *Pv*NLP3 Are Non-Cytotoxic

Necrosis-induction takes place at a specific target in dicot plants after signal peptide-mediated secretion of the NLP to the apoplastic side of the cell (Qutob et al., 2006; Lenarčič et al., 2017). Cabral and co-authors (2012) showed that the originally non-cytotoxic *Ha*NLP can gain a necrosis-inducing ability by swapping of certain N-terminal domains with a necrosis-inducing NLP from *P. sojae* (NLP*_PS_*). While the ligation of the signal peptide from NLP*_PS_* to the 5'-end of *Ha*NLP3 had no effect, the authors were able to create a cytotoxic construct by swapping the whole N-terminal domain ending at the second conserved cysteine residue, from NLP*_Ps_* with the corresponding region of *Ha*NLP3. Swapping of the same domains between cytotoxic *Pi*NPP1.1 and *Pv*NLP1, *Pv*NLP2 or *Pv*NLP3 had no such effect. A signal peptide usually enables the translocation of a secreted effector into the apoplast using the predominant secretion system consisting of endoplasmic reticulum and Golgi apparatus (Petre and Kamoun, 2014). To date, little is known about the secretion pathways in plant pathogenic fungi and oomycetes. Several studies report proteins that must be secreted non-classical, using pathways making a signal peptide dispensable (Ridout et al., 2006; Liu et al., 2014). *Pv*NLP1, *Pv*NLP2, and *Pv*NLP3 showed high scores during analysis with SecretomeP, suggesting a high probability for non-classical secretion of these proteins. However, using vacuum infiltration, recombinant proteins should be delivered directly to the apoplast. An additional transportation system for the effectors should therefore not be necessary for necrosis-induction. Nevertheless, even applied directly to the apoplast the tested *Pv*NLPs were not able to induce necrosis; neither in the susceptible *V. vinifera* cv. Mueller-Thurgau nor in three American wild *Vitis* species which develop a strong HR as a reaction to the infection with *P. viticola*. Both experiments show that the absence of a signal peptide is not the reason for the non-cytotoxic character of *Pv*NLP1, *Pv*NLP2, and *Pv*NLP3. Indeed, also the positive control *Pi*NPP1.1 was not able to induce necrosis in the leaf disks of *Vitis* spp., although half of the applied amount induced strong necrosis in *N. benthamiana* (**Figure 4**). This observation corresponds to the results of Chong and colleagues (2014) who transiently expressed *Pi*NPP1.1 in *V. vinifera* cv. Syrah and were also not able to observe necrosis. To our knowledge, there is no oomycete NLP which can induce necrosis in *Vitis*. Even though, successful infiltration into the leaf disks is visible by the naked eye, we cannot entirely exclude the possibility that the experiment did not work because of the vacuum infiltration method used. Since *P. viticola* depends on a living host, one may assume the possibility of a mechanism for the detoxification of NLPs in these plants. One way of necrosis suppression was described in cacao plants (*Theobroma cacao*) by the action of the plant-derived cystatin *Tc*CYS4 (Santana et al., 2014). The NLP *Mp*NEP2 from *Moniliophthora perniciosa* activates cysteine proteases in cacao plants and induces hypersensitive cell death. Cystatins on the other hand can modulate cysteine proteases. *Tc*CYS4 overexpression mutants of *N. benthamiana* showed clearly reduced sensitivity to necrosis formation by the transient expression of *Mp*NEP2 (Santana et al., 2014). Nevertheless, transient *Pi*NPP1.1 expression in *V. vinifera* leads to the induction of the sugar transporter *Vv*SWEET4, suggesting a recognition of this NLP in *V. vinifera* (Chong et al., 2014). Since membrane disruption is rather a specific process (Qutob et al., 2006; Lenarčič et al., 2017), another possibility besides suppression would be a missing target site for NLPs in *V. vinifera*.

### *Pv*NLPs Are Localized in the Cytoplasm When Expressed Inside Plant Cells

As mentioned in the introduction, different types of NLPs may have different target sites, which possibly may also lie inside the plant cell. Proteins often carry domains at their N- or C-terminus which influence their function or transport and consequently their subcellular localization (Crivat and Taraska, 2012). To exclude such alterations caused by the fused fluorescent proteins, each NLP was analyzed as C- or N- terminal labeled fusion protein. To reduce a possible effect of the fluorescent protein on the localization or false positive results by the formation of artifacts (Snapp, 2005) both, RFP- and GFP-fusions were analyzed. After expression in *N. benthamiana* all analyzed NLPs showed the same localization inside the cytoplasm as well as a possible association with the nucleus. No differences between RFP and GFP or N-terminal and C-terminal labeling could be detected. Even when the localization pattern of unbound GFP or RFP was equal to pattern of labeled *Pv*NLPs, western blot analyses did not reveal unbound fluorescent proteins in the corresponding leaf areas (results not shown). Since all localization experiments were carried out in *N. benthamiana* further experiments are necessary to confirm if this pattern corresponds to the natural distribution in *Vitis* spp.

Immunogold-labeling with an, although necrotic type 2, NLP from *Fusarium oxysporum* (NEP1) performed by Bae et al. (2006) showed a localization of this protein in the cytoplasm similar to our observations for *Pv*NLPs. An association with chloroplasts, mitochondria, endoplasmic reticulum or vacuole was not detected (Bae et al., 2006). Furthermore, it was reported that NLPs from *Botrytis cinerea* (*Bc*NLP) localize inside the nucleus and on the nuclear envelope, respectively (Schouten et al., 2008). If NLPs really enter the nucleus or if they are just located at the nuclear envelope was not finally clarified (Schouten et al., 2008). However, *Bc*NLPs were only detected on already collapsing tomato cells, suggesting that the binding is due to the membrane binding affinity of NLPs.

Like Schouten and colleagues (2008) we could not clarify if *Pv*NLPs are transported to the nucleus or just accumulated at the nuclear envelope in the cytoplasm. However, this should be further investigated by the use of confocal microscopy with suitable nuclear markers.

### *Pv*NLPs Might Dimerize in Solution

Pathogen secreted effectors often form oligomers which influences their function and recognition inside the plant cell. Effector SLP1 from *M. oryzae*, which suppresses the chitin triggered immune response in its host plant rice forms oligomers (Mentlak et al., 2012). Also many pore forming toxins undergo an oligomerization (Schoehn et al., 2003). The actinoporin Sticholysin II from the sea anemone *Stichodactyla helianthus* normally appears as a monomer. However, in the presence of a lipid membrane it forms tetramers, leading to a conformational change and consequently to pore formation in the membrane (Álvarez et al., 2009).

Also, several NLPs tend to dimer- or oligomerize. Two examples are *Mp*NEP1 from *M. perniciosa* and NPP1 from *P. parasitica,* which form homotrimers in solution (Garcia et al., 2007). Another NLP from *M. perniciosa, Mp*NEP2, forms metastable dimers in the host plant which leads to occlusion of the heptapeptide motif (de Oliveira et al., 2012). It is assumed that this mechanism suppresses the necrosis inducing activity of *Mp*NEP2 (de Oliveira et al., 2012). Alterations in environmental conditions like temperature changes might reverse dimerization and as a result lead to regulation of the necrosis inducing activity (de Oliveira et al., 2012).

Potential homo- or heterodimerization of *Pv*NLPs *in planta* was tested with co-immunoprecipitation assays using GFP- and RFP-tagged fusion proteins. Dimerization of the tested NLPs in *N. benthamiana* was not detectable. Since PvNLPs are relatively small, presence of the approximately equal sized fluorescence protein may inhibit dimerization. Nevertheless, recombinant *Pv*NLPs seem to form relatively stable homodimers in solution visible in Western blot analysis under strong denaturing conditions. However, future experiments, including native PAGE, must confirm dimerization of *Pv*NLPs and clarify its potential influence after a possible secretion from *P. viticola* into solution.

### Possible Functions of Non-Cytotoxic NLPs

Like other scientists, we assume a function of non-cytotoxic NLPs during the infection of host plants, however, their exact role still remains unknown (Cabral et al., 2012; Dong et al., 2012; Santhanam et al., 2013). NLPs show great structural similarities to lectins (Ottmann et al., 2009). Lectins are present in all kingdoms of life and are defined by their ability to bind carbohydrates as for example glucans (Chrispeels and Raikhel, 2007). Functions of lectins are diverse. Most of them are secreted into the cell wall and to the intercellular space, respectively (Chrispeels and Raikhel, 2007). One elicitor with lectin-like properties is for example the cellulose- binding elicitor lectin (CBEL) from *P. parasitica* var. *nicotianae*. CBEL induces necrosis and the expression of defense related genes after infiltration into tobacco leaves (Gaulin, 2002). Furthermore, it is involved in the adhesion of pathogen structures to cellulose, the main component of plant cell walls (Gaulin, 2002). One possible function of early expressed NLPs may therefore be an involvement in the process of zoospore attachment.

Another potential function of these proteins arises from the cell wall components of oomycetes. In addition to cellulose, the cell wall of these microorganisms consists of β-1-3-glucans. β-1-3-glucan binding effectors seem to be conserved over several kingdoms of life (Fesel and Zuccaro, 2016) and L-Typ receptor like kinases (LecRKs) are known to be involved in the resistance of several crops against pathogens (Singh and Zimmerli, 2013). NLPs might be able to bind β-1-3-glucanes due to their lectin like structure, and thereby suppress the recognition of these PAMPs during PTI. NLPs could thereby act as a virulence factor and extend the timeframe for a successful infection by the zoospores.

## Data Availability Statement

The sequencing data has been uploaded to GenBank. DNA sequences of PvNLP1 and PvNLP3 can be found using accession numbers MN564834 and MN720268. The polymorphisms found during sequencing (**Supplemental Figures 2**–**4**) can be found using accession numbers MN938410, MN938411 and MN938412.

## Author Contributions

RF, H-HK, SS, and RV conceived the project and designed the experiments. KG and SS performed the different experiments. RF, KG, and SS processed all data and did the statistical and bioinformatics analyses. SS wrote the manuscript and all co-authors read and approved it.

## Funding

This work was co-financed by the European Union—European Regional Development Fund as a part of the BACCHUS Interreg project IV Oberrhein/Rhin supérieur and Vitifutur Interreg project V Oberrhein/Rhin supérieur.

## Conflict of Interest

The authors declare that the research was conducted in the absence of any commercial or financial relationships that could be construed as a potential conflict of interest.
