# Supplementary material for: Identification and Characterization of Nep1-Like Proteins From the Grapevine Downy Mildew Pathogen Plasmopara viticola
Source: Front Plant Sci. 2020 Feb 13;11:65. doi: 10.3389/fpls.2020.00065 (PMC7031652; doi:10.3389/fpls.2020.00065)
Supplement: Supplementary file 1 [file DataSheet_1.pdf]

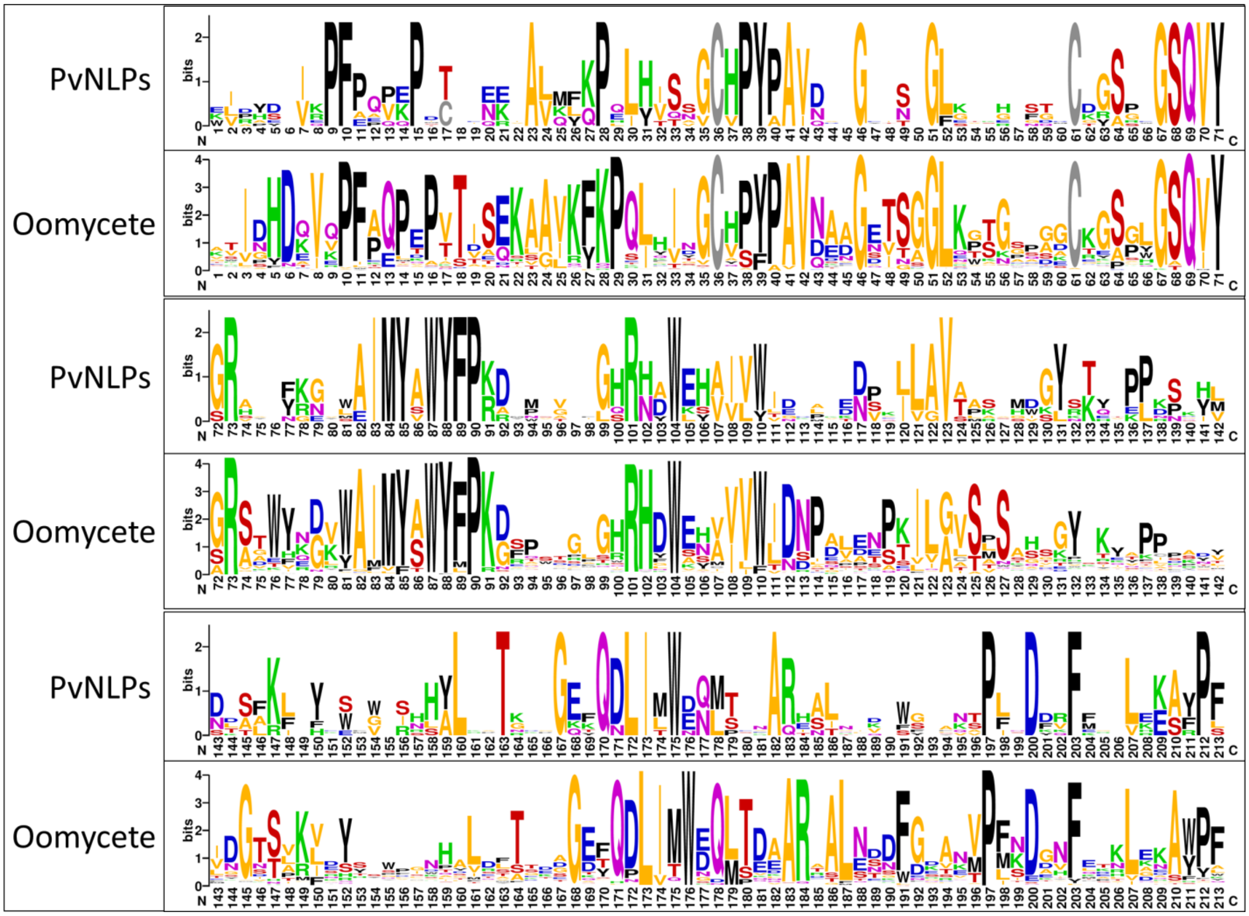

Supplemental Figure 1: Comparison of *Pv*NLPs with all oomycete NLPs:

Aligned Weblogos of eight *Pv*NLP sequences and 230 oomycete NLP sequences obtained from Oome and Van den Ackerveken (2014).
