# Supplementary material for: Identification and Characterization of Nep1-Like Proteins From the Grapevine Downy Mildew Pathogen Plasmopara viticola
Source: Front Plant Sci. 2020 Feb 13;11:65. doi: 10.3389/fpls.2020.00065 (PMC7031652; doi:10.3389/fpls.2020.00065)
Supplement: Supplementary file 2 [file DataSheet_2.pdf]

```
Pv1306_PvNLP1     1  MSPWEAKWIRHSDVRPFPQPEPMTVEEKVAVMLKPELHLSSGCHPYPAVNDLGETNGGLK
PvNLP1            1  MSPWEAKWIRHSDVRPFPQPEPMTVEEKVAVMLKPELHVSSGCHPYPAVNDLGETNGGLK


Pv1306_PvNLP1    61  TTGAPSGMCKGSGWGSQFTVDTHHLEASGPSCTRGTFQKTCRRHTSGIATTGST
PvNLP1           61  TTGAPSGMCKGSGWGSQFTVDTHHLEASGPSCTRGTFQKTCRRHTSGIATTGST
```

Supplemental Figure 2: Amino acids substitution in *Pv*NLP1 from isolate *Pv*1306

Grey color indicates amino acids with the same chemical properties.
