# Supplementary material for: Identification and Characterization of Nep1-Like Proteins From the Grapevine Downy Mildew Pathogen Plasmopara viticola
Source: Front Plant Sci. 2020 Feb 13;11:65. doi: 10.3389/fpls.2020.00065 (PMC7031652; doi:10.3389/fpls.2020.00065)
Supplement: Supplementary file 3 [file DataSheet_3.pdf]

```
Pv1446_PvNLP2    1 MSPWEAKWIRHSEVRPFPQPEPITVEEKVAVMLKPEIHVKNGCHPYPAVNDLGETNSGLK
PvNLP2           1 MSPWEAKWIRHSEVRPFPQPEPITVEEKVAVMLKPEIHVTNGCHPYPAVNDLGETNSGLK

Pv1446_PvNLP2   61 TKGAPSGMCKGSGWGSQVYGRHALFKGVWAIMYSWYFPKDMPSTDFGHRHDWEHVIVWIE
PvNLP2          61 TKGAPSGMCKGSGWGSQVYGRHALFKGVWAIMYSWYFPKDMPSTDFGHRHDWEHVIVWIE

Pv1446_PvNLP2  121 KPVVENVKILAVTPSAHDGYSKQVPPNPGHLNGLAAKINYESKWPINHALEPTGLGGEKQ
PvNLP2         121 KPVVENVKILAVTPSAHDGYSKQVPPNPGHLNGLAAKINYESKWPINHALEPTGLGGEKQ

Pv1446_PvNLP2  181 DLILWEQLSSNARHALNIVHWGDANTPFNDYVFMGKLEKAFPL
PvNLP2         181 DLILWEQLSSNARHALNIVHWGDANTPFNDYVFMGKLEKAFPL
```

Supplemental Figure 3: Amino acid substitution in *Pv*NLP2 from isolate *Pv*1446
