# Supplementary material for: Identification and Characterization of Nep1-Like Proteins From the Grapevine Downy Mildew Pathogen Plasmopara viticola
Source: Front Plant Sci. 2020 Feb 13;11:65. doi: 10.3389/fpls.2020.00065 (PMC7031652; doi:10.3389/fpls.2020.00065)
Supplement: Supplementary file 4 [file DataSheet_4.pdf]

```
1137-C20_PvNLP3    1  MGPWEAKWIRHSDVRPFPQPEPMTVEEKVAVMLKPELHVSSGCHPYPAVNDLGETNGGLK
Pv1252_PvNLP3      1  MGPWEAKWIRHSDVRPFPQPEPMTVEEKVAVMLKPELHVSSGCHPYPAVNDLGETNGGLK
Pv1366_PvNLP3      1  MGPWEAKWIRHSDVRPFPQPEPMTVEEKVAVMLKPELHVSSGCHPYPAVNDLGETNGGLK
Pv1379_PvNLP3      1  MGPWEAKWIRHSDVRPFPQPEPMTVEEKVAVMLKPELHVSSGCHPYPAVNDLGETNGGLK
PvBr13_PvNLP3      1  MGPWEAKWIRHSDVRPFPQPEPMTVEEKVAVMLKPELHVSSGCHPYPAVNDLGETNGGLK
PvNLP3             1  MGPWEAKWIRHSDVRPFPQPEPMTVEEKVAVMLKPELHVSSGCHPYPAVNDLGETNGGLK

1137-C20_PvNLP3   61  TTGAPSGMCKGSGWGSQIYGRHASFRGVWAIMYVWYFPKDMPSAHFGHRHDWEHVIVWIE
Pv1252_PvNLP3     61  TTGAPSGMCKGSGWGSQIYGRHASFRGVWAIMYVWYFPKDMPSAHFGHRHDWEHVIVWIE
Pv1366_PvNLP3     61  TTGAPSGMCKGSGWGSQIYGRHASFRGVWAIMYVWYFPKDMPSAHFGHRHDWEHVIVWIE
Pv1379_PvNLP3     61  TTGAPSGMCKGSGWGSQIYGRHASFRGVWAIMYVWYFPKDMPSAHFGHRHDWEHVIVWIE
PvBr13_PvNLP3     61  TTGAPSGMCKGSGWGSQIYGRHASFRGVWAIMYVWYFPKDMPSAHFGHRHDWEHVIVWIE
PvNLP3            61  TTGAPSGMCKGSGWGSQVYGRHASFRGVWAIMYVWYFPKDMPSAHFGHRHDWEHVIVWIE

1137-C20_PvNLP3  121  KPVVENVKILAVTPSFHDGYSKQVPPDPSHLNGLAAKFIYESEWPINHALRPTRKGGKKQ
Pv1252_PvNLP3    121  KPVVENVKILAVTPSFHDGYSKQVPPDPSHLNGLAAKFIYESEWPINHALRPTRKGGKKQ
Pv1366_PvNLP3    121  KPVVENVKILAVTPSFHDGYSKQVPPDPSHLNGLAAKFIYESEWPINHALRPTRKGGKKQ
Pv1379_PvNLP3    121  KPVVENVKILAVTPSFHDGYSKQVPPDPSHLNGLAAKFIYESEWPINHALRPTRKGGKKQ
PvBr13_PvNLP3    121  KPVVENVKILAVTPSFHDGYSKQVPPDPSHLNGLAAKFIYESEWPINHALRPTRKGGKKQ
PvNLP3           121  KPVVENVKILAVTPSFHDGYSKQVPPDPSHLNGLAAKFIYESEWPINHALRPTRKGGKKQ

1137-C20_PvNLP3  181  DLILWEQMSSNARHALNIVPWGAANTPFNDFVFMGRLEKAFPF
Pv1252_PvNLP3    181  DLILWEQMSSNARHALNIVPWGAANTPFNDFVFMGRLEKAFPF
Pv1366_PvNLP3    181  DLILWEQMSSNARHALNIVPWGAANTPFNDFVFMGRLEKAFPF
Pv1379_PvNLP3    181  DLILWEQMSSNARHALNIVPWGAANTPFNDFVFMGRLEKAFPF
PvBr13_PvNLP3    181  DLILWEQMSSNARHALNIVPWGAANTPFNDFVFMGRLEKAFPF
PvNLP3           181  DLILWEQMSSNARHALNIVPWGAANTPFNDFVFMGRLEKAFPF
```

Supplemental Figure 4: Amino acid substitution in *Pv*NLP3 from the isolates *Pv*1252, *Pv*1366, *Pv*1379, *Pv*Br13 and the single sporangia line 1137-C20

Grey color indicates amino acids with similar chemical properties.
