# Supplementary material for: Identification and Characterization of Nep1-Like Proteins From the Grapevine Downy Mildew Pathogen Plasmopara viticola
Source: Front Plant Sci. 2020 Feb 13;11:65. doi: 10.3389/fpls.2020.00065 (PMC7031652; doi:10.3389/fpls.2020.00065)
Supplement: Supplementary file 5 [file DataSheet_5.pdf]

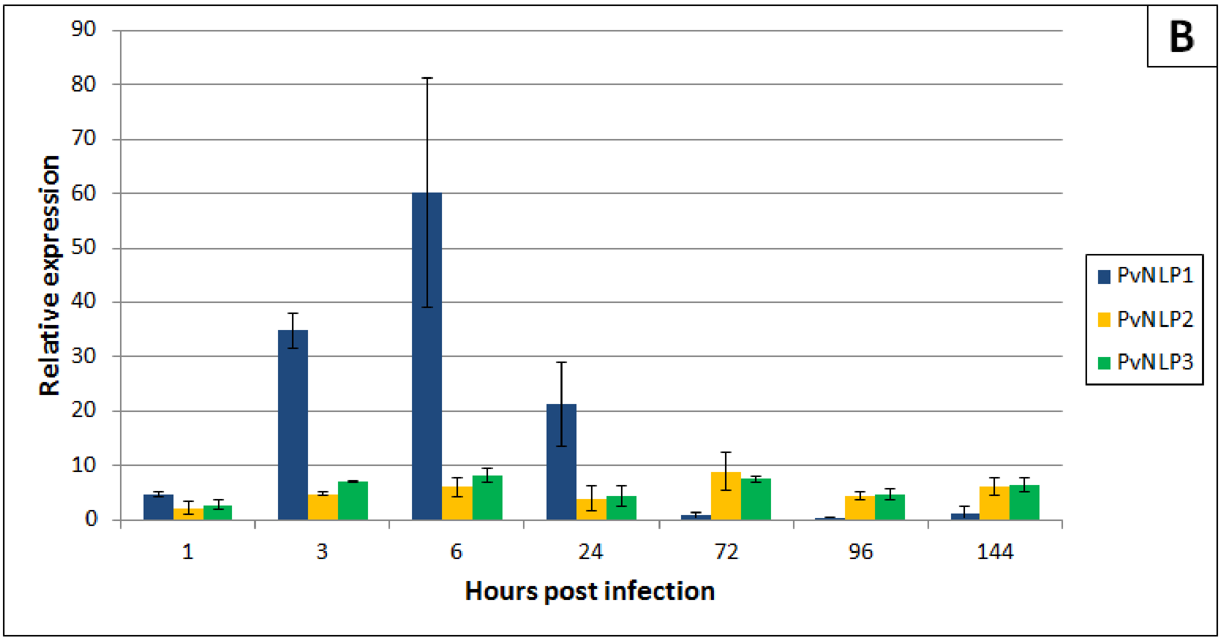
B
Relative expression
0
10
20
30
40
50
60
70
80
90
1
3
6
24
72
96
144
Hours post infection
PvNLP1
PvNLP2
PvNLP3

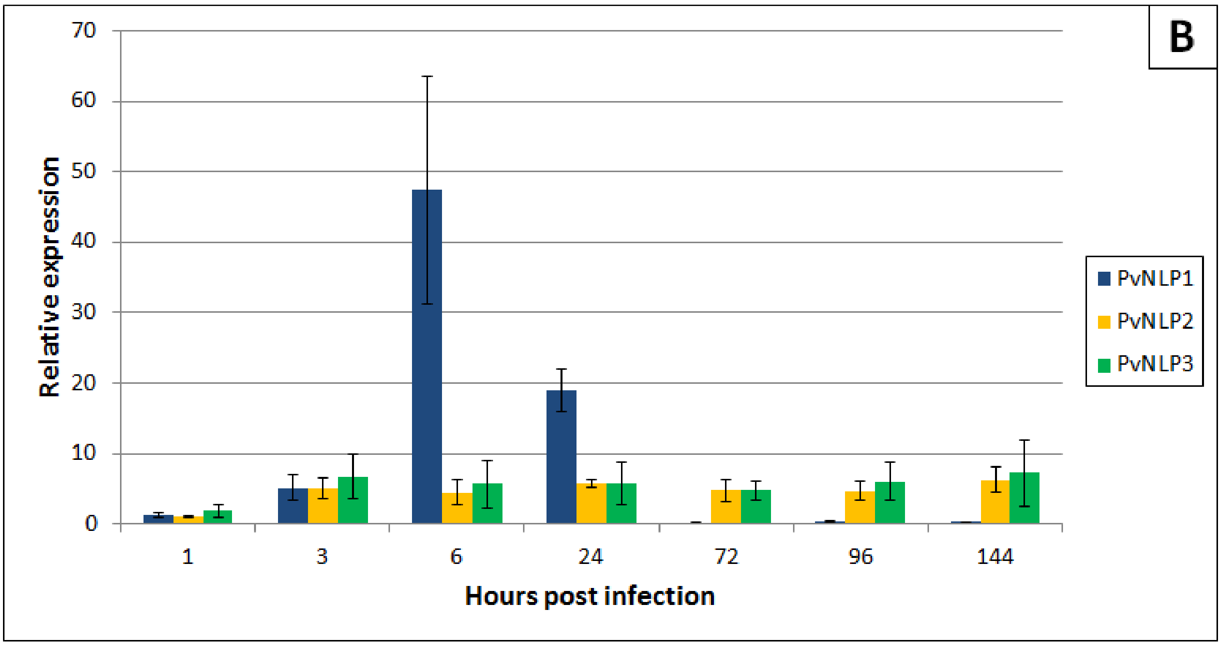
B
Relative expression
0
10
20
30
40
50
60
70
1
3
6
24
72
96
144
Hours post infection
PvNLP1
PvNLP2
PvNLP3

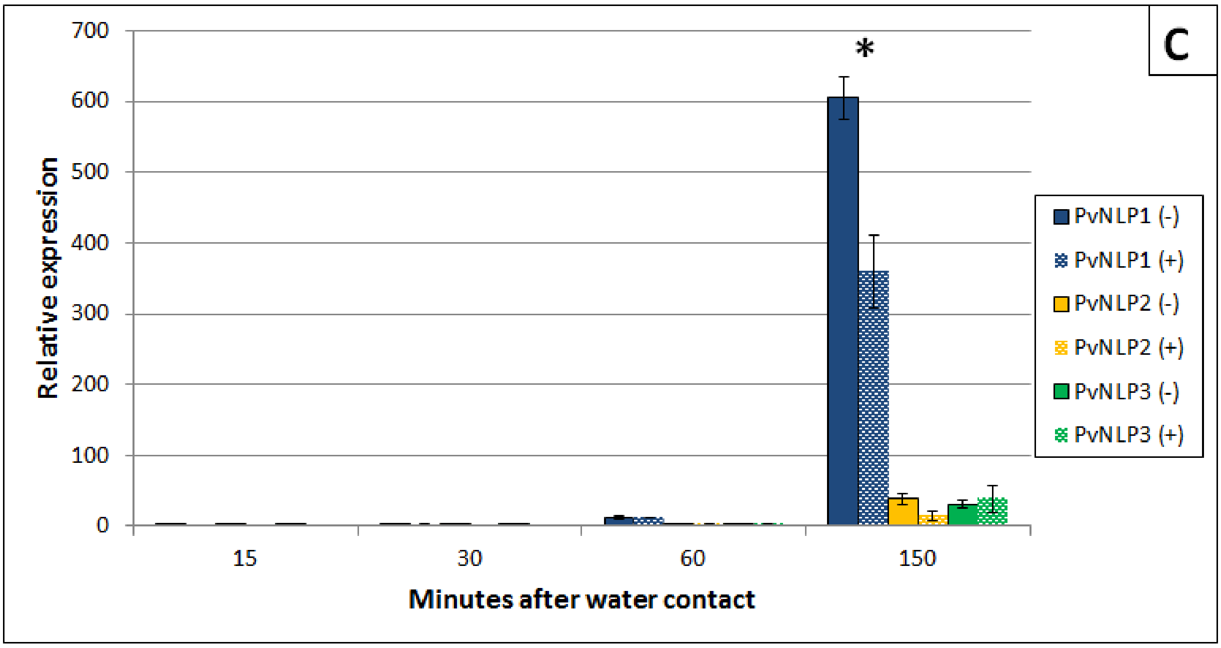

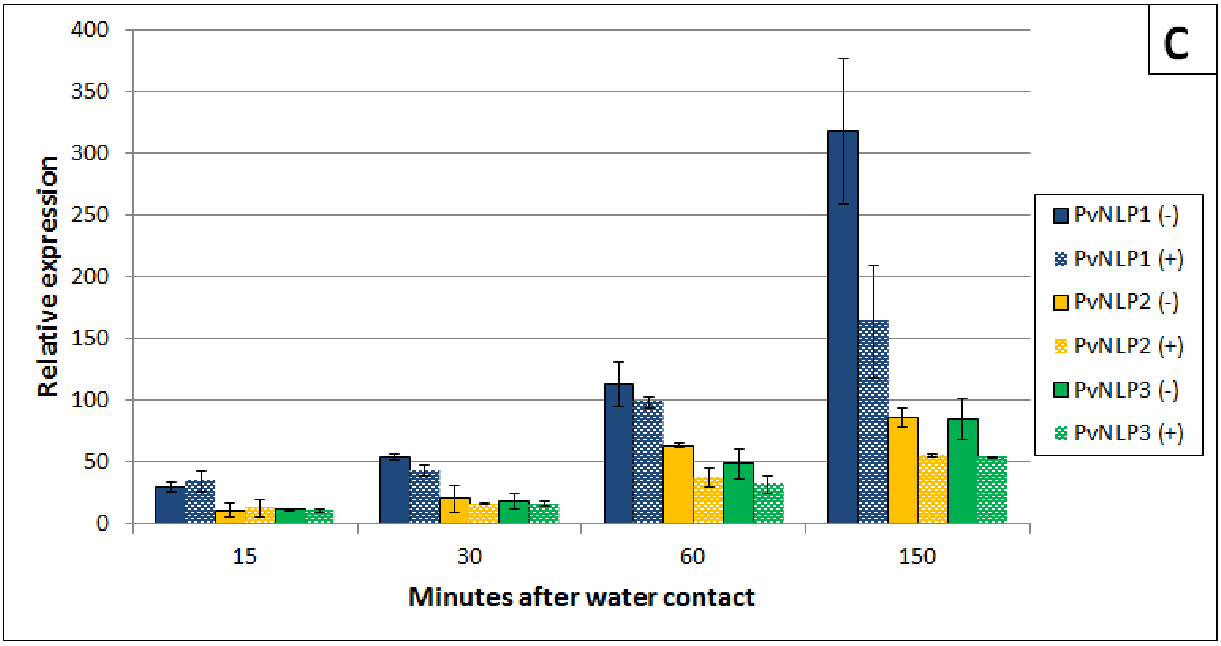

Supplemental Figure 5: Expression of *P. viticola NLPs* 1 – 3 during infection of *V. vinifera* cv. Mueller-Thurgau

B: Relative expression of *Pv*NLP1, *Pv*NLP2 and *Pv*NLP3 on infected *V. vinifera* cv. Mueller-Thurgau leaf disks at the time points: zoospore release (1 h), penetration (3 h), first haustoria visible (6 h), branching of hyphae (24 h), 72h, 96h, and 144 h after inoculation. Each graph

represents one of three experiments with similar results. Bars are the mean of three technical replicates ± SD. C: Relative expression of *PvNLP*1, *PvNLP2* and *PvNLP3* in a sporangia solution with (+) or without (-) presence of leaf material at the time points 15, 30, 60 (beginning of zoospore release from the sporangia) and 150 minutes after water contact. Each graph represents one of three experiments with similar results. Bars are the mean of three technical replicates ± SD. Asterisk indicates significance ($p \leq 0.05$, one-way Anova)
