# Supplementary material for: Identification and Characterization of Nep1-Like Proteins From the Grapevine Downy Mildew Pathogen Plasmopara viticola
Source: Front Plant Sci. 2020 Feb 13;11:65. doi: 10.3389/fpls.2020.00065 (PMC7031652; doi:10.3389/fpls.2020.00065)
Supplement: Supplementary file 6 [file DataSheet_6.pdf]

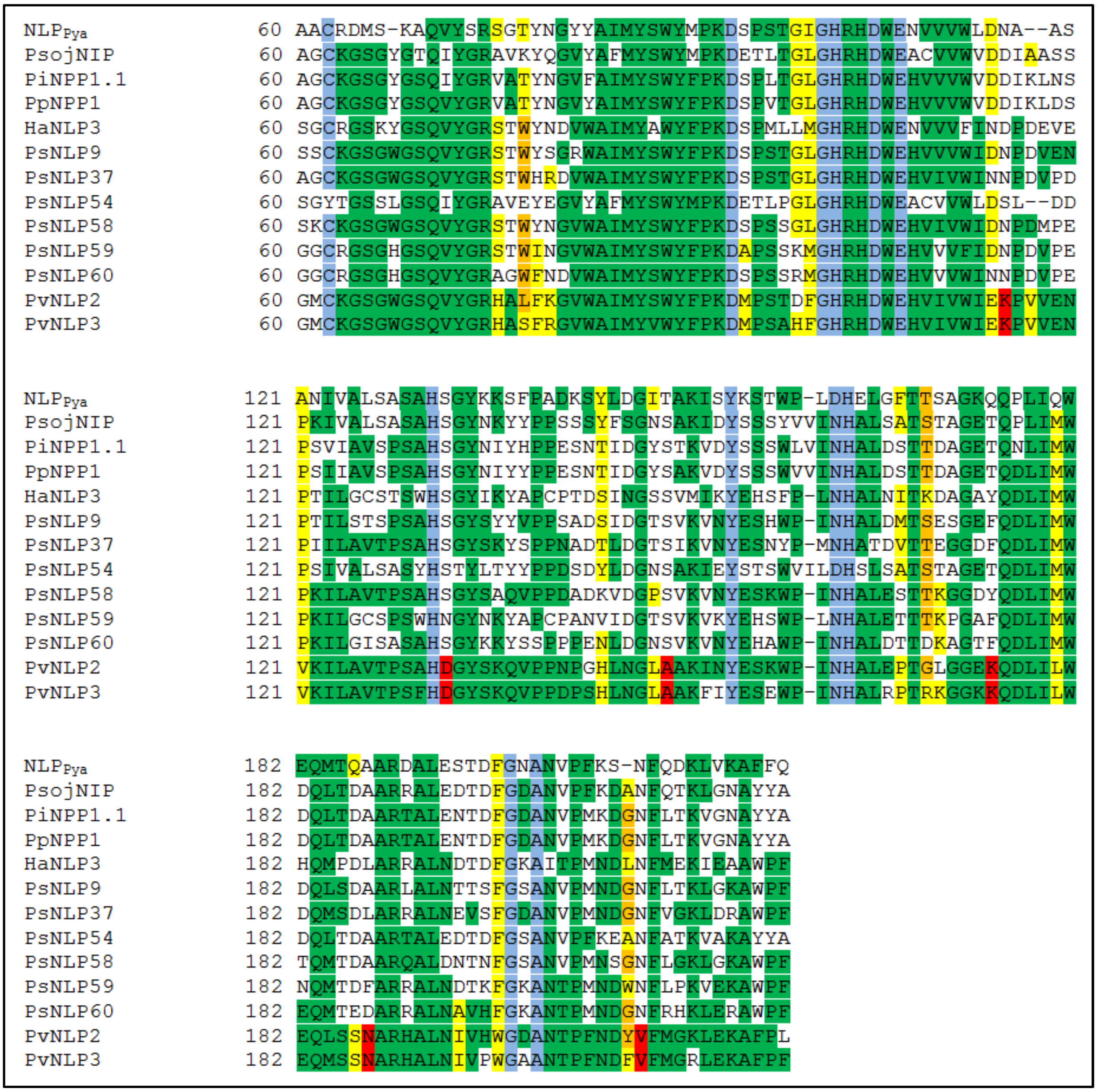

Supplemental Figure 6: Alignment of C-termini from cytotoxic NLPs and PvNLPs

Green color indicates positions in the sequence where at least PvNLP2 or PvNLP3 shares an identical amino acid with one of the NLPs above; yellow/orange color indicates positions in the sequence where at least PvNLP2 or PvNLP3 shares an amino acid with similar chemical properties. Blue color indicates amino acids described as crucial for necrosis induction in several publications (Oome and Van den Ackerveken, 2014; Ottmann et al., 2009). Red color indicates positions of amino acids which are completely different in PvNLP2 and PvNLP3 compared to the other NLPs in the alignment.
